# HSD17B4, ACAA1, and PXMP4 in Peroxisome Pathway Are Down-Regulated and Have Clinical Significance in Non-small Cell Lung Cancer

**DOI:** 10.3389/fgene.2020.00273

**Published:** 2020-03-20

**Authors:** Xiuzhi Zhang, Hongmei Yang, Jinzhong Zhang, Fenglan Gao, Liping Dai

**Affiliations:** ^1^Department of Pathology, Henan Medical College, Zhengzhou, China; ^2^Institute of Cancer Research, Henan Medical College, Zhengzhou, China; ^3^Henan Institute of Medical and Pharmaceutical Sciences, Zhengzhou University, Zhengzhou, China

**Keywords:** lung cancer, peroxisome, prognosis, diagnosis, anti-cancer drug sensitivity

## Abstract

To explore the potential functions and clinical significances of peroxisomes during lung cancer development and progression, we investigated the expressional profiles of peroxisome pathway genes and their correlations with clinical features in non-small cell lung cancer (NSCLC). The RNA-seq data of NSCLC including lung squamous carcinoma (LUSC) and lung adenocarcinoma (LUAD) patients with their clinical information were downloaded from The Cancer Genome Atlas (TCGA). Gene expression comparisons between tumor and normal samples were performed with edgeR package in R software and the results of the 83 peroxisome pathway genes were extracted. Through Venn diagram analysis, 38 common differentially expressed peroxisome pathway genes (C-DEPGs) in NSCLC were identified. Principal components analysis (PCA) was performed and the 38 C-DEPGs could discriminate NSCLC tumors from the non-tumor controls well. Through Kaplan-Meier survival and Cox regression analyses, 11 of the C-DEPGs were shown to have prognostic effects on NSCLC overall survival (OS) and were considered as key C-DEPGs (K-DEPGs). Through Oncomine, Human Protein Atlas (HPA) and the Clinical Proteomic Tumor Analysis Consortium (CPTAC), three K-DEPGs (HSD17B4, ACAA1, and PXMP4) were confirmed to be down-regulated in NSCLC at both mRNA and protein level. Their dy-regulation mechanisms were revealed through their correlations with their copy number variations and methylation status. Their potential functions in NSCLC were explored through their NSCLC-specific co-expression network analysis, their correlations with immune infiltrations, immunomodulator gene expressions, MKI67 expression and their associations with anti-cancer drug sensitivity. Our findings suggested that HSD17B4, ACAA1, and PXMP4 might be new markers for NSCLC diagnosis and prognosis and might provide new clues for NSCLC treatment.

## Introduction

Lung cancer is the most frequently diagnosed cancer and the leading cause of cancer death worldwide, with about 2.1 million new lung cancer cases and 1.8 million lung cancer deaths every year (Bray et al., 2018). Almost 80–85% of the lung cancer cases were non-small cell lung cancer (NSCLC) which included two major histological types: lung squamous carcinoma (LUSC) and lung adenocarcinoma (LUAD) (Travis et al., 2015). For the NSCLC patients at an early stage (stage I–II), surgery is the recommended treatment and the 5-year survival is about 53–92% (Vansteenkiste et al., 2014; Goldstraw et al., 2016). However, for the patients at a locally advanced or metastatic stage (stage III or IV), the operation is not amendable and the 5-year-survival is only 4–20% (Siegel et al., 2017; Osmani et al., 2018). To improve the lung cancer outcomes, it is crucial to find new markers for its early diagnosis and prognostic predication.

Peroxisomes are ubiquitous cellular organelles which can be found in nearly all eukaryotic cells. They were first described by Johannes Rhodin in 1954 and termed as “microbodies.” As there were a lot of hydrogen peroxide metabolizing enzymes in the microbodies, they were then called “peroxisomes” functionally (De Duve and Baudhuin, 1966). In fact, besides involving in the synthesis and turnover of reactive oxygen species (ROS) (Bonekamp et al., 2009; Antonenkov et al., 2010), the peroxisomes also play important roles in fatty acid ɑ- and/or β-oxidation (Poirier et al., 2006; Wanders and Waterham, 2006), the catabolism of purines (Sprecher et al., 1995) and the biosynthesis of glycerolipids (Wanders et al., 2010) which are indispensable to human health and development. Metabolic reprogramming is one of the core traits of the cancer cells, due to the multiple alterations during the multi-step process of tumorigenesis (Soga, 2013; El Hassouni et al., 2019). In recent years, with the deepening understanding of the important functions of the peroxisomes, more and more attention were paid to the peroxisomes research (Tanner et al., 2013; El Hassouni et al., 2019) and they were demonstrated to be implicated in innate immunity (Ferreira et al., 2019), signal transduction (Schrul and Schliebs, 2018), aging (Deori et al., 2018), and cancer (Dahabieh et al., 2018).

In fact, dy-regulations of peroxisomal enzymes and/or their effects were shown in numerous tumor types including prostate cancer (Ko et al., 2018), colorectal carcinoma (Lakis et al., 2010), liver cancer (Lu et al., 2015; Chen et al., 2018), oral squamous cell carcinoma (Lai et al., 2018), pancreatic cancer (Deplanque et al., 2015), breast cancer (Keller et al., 1993), and lymphoma (Zheng et al., 2019). However, there was no systemic study of peroxisomes in lung cancer. Here, we identified 38 common differentially expressed genes in peroxisome pathway in NSCLC from The Cancer Genome Atlas (TCGA) project and evaluated their prognostic effects in LUSC and LUAD, respectively. Interestingly, 11 of the genes were shown to have prognostic effects but only in LUSC or LUAD individually. However, three of them were confirmed to be consistently under-expressed in LUSC and LUAD at both mRNA and protein level. We then constructed the NSCLC-specific co-expression network of the three genes, explored their potential functions, evaluated their correlations with the immune infiltrations, immunomodulator gene expressions, proliferation marker (MKI67) expression and the sensitivities of anti-cancer drugs in NSCLC. These results might provide new clues for the values of peroxisomes in NSCLC diagnosis, prognosis and treatment.

## Materials and Methods

### Available Data From TCGA Database

The RNA-seq data of 501 TCGA-LUSC patients (including 501 primary tumor and 49 normal tissue samples) and 513 TCGA-LUAD patients (including 513 primary tumor and 59 normal tissue samples) with their clinical information were downloaded from Genomic Data Commons (GDC) data portal^1^. The clinical features of the patients were listed in Table 1.

**TABLE 1:** Clinical features of TCGA-LUSC and TCGA-LUAD patients.

Clinical features	LUSC (*n* = 501)	LUAD (*n* = 513)
**Age (year)**		
≤60	108(21.6%)	157(30.6%)
>60	384(76.6%)	337(65.7%)
NA	9(1.8%)	19(3.7%)

**Sex**	***n* (%)**	***n* (%)**

Male	371(74.1%)	237(46.2%)
Female	130(25.9%)	276(53.8%)

**TNM stage**	***n* (%)**	***n* (%)**

Stage I	244(48.7%)	274(52.8%)
Stage II	159(31.7%)	121(23.6%)
Stage III	84(16.8%)	84(16.4%)
Stage IV	7(1.4%)	26(5.1%)
NA	7(1.4%)	8(1.6%)

**Race**	***n* (%)**	***n* (%)**

White	349(69.7%)	387(75.4%)
Black or African American	30(6.0%)	52(10.1%)
Asian	9(1.8%)	7(1.4%)
American Indian or Alaska Native	0(0%)	1(0.2%)
NA	113(22.5%)	66(12.9%)

**Survival status**	***n* (%)**	***n* (%)**

Alive	285(56.9%)	321(62.6%)
Dead	210(41.9%)	183(35.7%)
NA	6(1.2%)	9(1.7%)

*LUSC, lung squamous carcinoma; LUAD, lung adenocarcinoma; NA, not available.*

### Differential Expression Analysis and Prognostic Effects Evaluation of Peroxisome Pathway Genes in NSCLC From TCGA Database

The information of the peroxisome pathway genes (*n* = 83) was investigated with KEGG database^2^. EdgeR package in R software (R3.5.2) was used for expressional comparisons of the genes between tumor and normal tissues in TCGA-LUSC and TCGA-LUAD datasets and the expressional differences of the peroxisome pathway genes were extracted. The genes with false discovery rate (FDR) < 0.01 were considered to be statistically significant differentially expressed peroxisome pathway genes (DEPGs). The intersection of the two sets of the DEPGs in LUSC and LUAD were considered common DEPGs (C-DEPGs) which were consistently up- or down-regulated in the two subtypes. Principal components analysis (PCA), which was applied widely for effective dimension reduction and exploratory visualization, was confirmed to be useful to correct the possibility of false association and show the difference between case and control clearly (Price et al., 2006; Zhang and Castello, 2017). In this study, through GEPIA^3^, PCA was performed to evaluate the discriminating power of the C-EDPGs in differentiating NSCLC from non-tumor lung tissues. In GEPIA, the Genotype-Tissue expression (GTEx) normal data was used to solve the imbalance between the tumor and normal data which can cause inefficiency in various differential analyses and the TCGA and GTEx gene expression data were all Trans Per Million (TPM) normalized from the raw RNA-Seq data by the UCSC Xena project based on a uniform pipeline (Tang et al., 2017).

To evaluate the prognostic effects of the C-DEPGs on overall survival (OS) of the NSCLC patients, with SPSS 18.0, Kaplan-Meier survival analysis with log rank test was performed in LUSC and LUAD, respectively, and the hazard ratios (HRs) were obtained from univariate Cox proportional hazard models. For the above analyses, the median expression of each gene was set as the threshold and the patients were divided into low expression and high expression groups. The genes with significant prognostic effects (*p* < 0.05) were considered as the key C-DEPGs (K-DEPGs).

### Validations of the Expressional Differences of the K-DEPGs in NSCLC

At mRNA level, the K-DEPGs were compared between tumor and normal lung tissues in other LUSC and LUAD datasets via Oncomine database. For the comparisons, the filters were used as follows: analysis type: lung adenocarcinoma vs. normal analysis, squamous cell lung carcinoma vs. normal analysis; data type: mRNA; *p*-value: 0.05. At protein level, the immunohistochemical (IHC) data of the K-DEPGs in lung cancer and normal lung tissues were investigated from the Human Protein Atlas (HPA) database and the results were validated by the lung cancer proteomic data from Clinical Proteomic Tumor Analysis Consortium (CPTAC)^4^ which included 111 unique LUAD tumor samples and 102 unique normal samples analyzed by global proteomic mass spectrometry using the 10-plexed isobaric tandem mass tags (TMT-10) following the CPTAC reproducible workflow protocol. The clinical features of the lung cancer dataset from CPTAC were shown in Supplementary Table S1. The expressional differences of the proteins between early stage (stage I) and late stage (stage II/stage III/stage IV) and between well/moderate differentiation (grade 1/grade 2) and poorly differentiation (grade 3) were also evaluated to investigated their associations with NSCLC progression. The non-parametric, two-independent-samples Wilcoxon test was used to evaluate the protein differences and *p* < 0.05 was considered significant.

### Influences of Copy Number Variations and Methylation Values on the Expressions of the Confirmed Genes in NSCLC

To further uncover the potential mechanisms of the dy-regulation of the confirmed genes, their correlations with copy number variations (CNVs) in TCGA-LUSC and TCGA-LUAD were analyzed through cBioPortal^5^, a publicly accessible resource providing visualization and analysis tools for more than 5,000 tumor samples from 232 cancer studies in the TCGA pipeline. The correlations between the methylation status of the genes and their expressions were investigated via MEXPRESS^6^, an online tool for visualization of DNA methylation and expression data from TCGA. Spearman’s correlation and Pearson’s correlation were used for the analyses and the absolute value of correlation coefficient >0.1 with *p* < 10^–5^ was considered significant.

### NSCLC Specific Co-expression Network Construction of the Confirmed K-DEPGs

Co-expressed genes were shown to be involved in similar biological processes and functionally related (Stuart et al., 2003). Co-expression network analysis was confirmed to be effective to explore new functions for specific genes (Zhong et al., 2017; Dugo et al., 2018). Here, The NSCLC specific co-expression network of the three confirmed K-DEPGs including HSD17B4, ACAA1, and PXMP4 were constructed through a database for tissue and cancer specific biological networks (TCSBN)^7^. The co-expressed genes in the network were then analyzed through Metascape^8^ to explore the potential functions of the three confirmed genes during NSCLC development.

### Further Insight of Potential Roles of the Confirmed K-DEPGs in NSCLC Immunoregulation and Tumor Proliferation

The abundances of six immune infiltrates (B cells, CD4^+^ T cells, CD8^+^ T cells, Neutrophils, Macrophages and Dendritic cells) in LUSC and LUAD were investigated Via TIMER^9^, a comprehensive resource for systematical analysis of immune infiltrates across diverse cancer types. The immunomodulator genes (*n* = 91) including 24 immunoinhibitors, 46 immunostimulators, and 21 major histocompatibility complex (MHC) related genes were downloaded from The Cancer Immunome Atlas (TCIA)^10^. To investigate their associations with immune processes, the confirmed C-DEPGs (HSD17B4, ACAA1, and PXMP4) expressions were analyzed for their purity-corrected Spearman correlations with the immune infiltrations and the immunomodulator gene expressions in NSCLC through TIMER.

MKI67, also known as Ki-67 protein, is expressed in all the proliferating cells and widely used as a proliferation marker (Gerdes et al., 1983; Scholzen and Gerdes, 2000; Booth et al., 2014). MKI67 expression profiles in NSCLC were investigated through TIMER at mRNA level and through HPA and CPTAC at protein level. To evaluate the potential roles of HSD17B4, ACAA1, and PXMP4 in NSCLC proliferation, their correlations with MKI67 expression were also evaluated both at mRNA level and protein level through TIMER and CPTAC, respectively. Wilcoxon test and Spearman correlation analysis was used for expressional comparisons and correlation evaluation, respectively. For all the analyses, *p* < 0.01 was considered statistically significant.

### Further Insights of the Potential Effects of the Confirmed K-DEPGs on Anti-cancer Drug Sensitivity/Resistance in NSCLC Cell Lines

The relative expressions of HSD17B4, ACAA1, and HSD17B4 in NSCLC cell lines and the pharmacologic profiles for 24 anti-cancer drugs across 504 cell lines were downloaded from Cancer Cell Line Encyclopedia (CCLE) database^11^. The half maximal inhibitory concentrations (IC50) of 24 anti-cancer drugs in 89 NSCLC cell lines were extracted. Spearman’s correlation analysis was also applied to investigate the associations between the three gene expressions and the IC50 values of the drugs in the cell lines. In addition, for each drug, the NSCLC cell lines were divided into sensitive group and resistant group with the drug IC50 of 8 μM as the threshold according to a previous study (Xiang et al., 2019) and the expressional differences of the three genes between the two groups were evaluated with Mann-Whitney U tests. The analyses were performed through SPSS 18.0 and *p* < 0.05 was considered significant.

## Results

### Identification of C-DEPGs Between Tumor and Normal Samples in NSCLC

**FIGURE 1:**
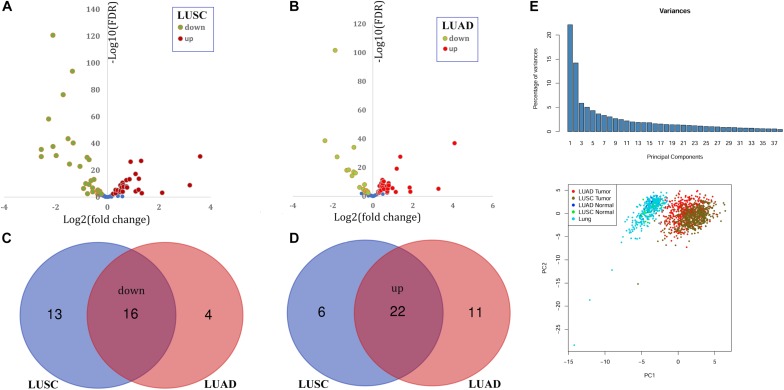
Identification and expression profiles of common differentially expressed peroxisome pathway genes (C-DEPGs) in NSCLC. **(A)** 57 DEPGs including 29 down-regulated and 28 up-regulated genes in LUSC. **(B)** 53 DEPGs including 20 down-regulated and 33 up-regulated genes in LUAD. **(C)** Venn diagram of the overlapping down-regulated DEPGs in LUSC and LUAD (down-regulated C-DEPGs in NSCLC). **(D)** Venn diagram of the overlapping up-regulated DEPGs in LUSC and LUAD (up-regulated C-DEPGs in NSCLC). **(E)** Two-dimensional principal component analysis (PCA) of gene expression profiles of the C-DEPGs. Each sample is represented with a single point, with different color for each of the groups. NSCLC, non-small cell lung cancer; LUSC, lung squamous carcinoma; LUAD, lung adenocarcinoma. LUAD tumor and LUSC tumor represented tumor samples in TCGA-LUAD dataset and TCGA-LUSC dataset, respectively. LUAD normal and LUSC Normal represented normal lung samples in TCGA-LUAD dataset and TCGA-LUSC dataset, respectively. Lung represented normal lung samples from The Genotype-Tissue Expression (GTEx) project.

With the filter FDR < 0.01, there were 57 and 53 DEPGs in LUSC and LUAD, respectively (Figures 1A,B). Among them, 38 genes including 16 down-regulated (Figure 1C) and 22 up-regulated genes (Figure 1D) were consistently differentially expressed in LUSC and LUAD and considered as C-DEPGs. Upon the PCA analysis through GEPIA (Figure 1E), the 38 C-DEPGs could discriminate the NSCLC tumors and non-tumor tissues effectively, indicating their diagnostic power in differentiating NSCLC and normal controls.

### Prognostic Effects of C-DEPGs

**FIGURE 2:**
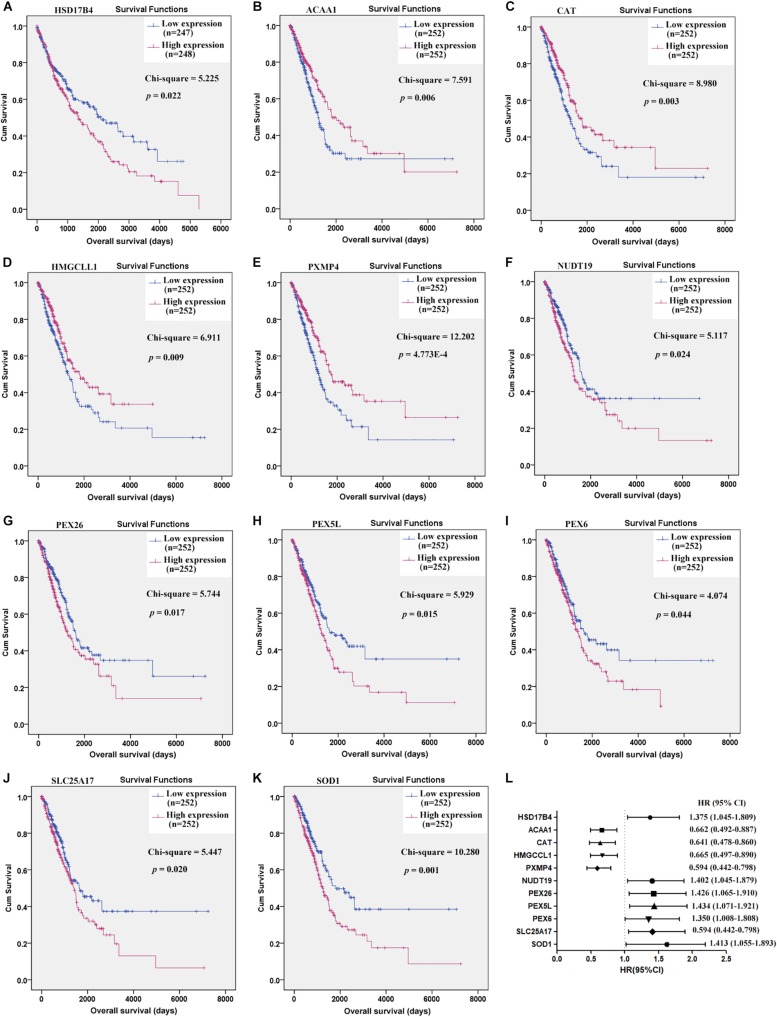
Prognostic effects of the key C-DEPGs (K-DEPGs) on NSCLC overall survival (OS). **(A)** Higher expression of HSD17B4 indicated shorter OS in LUSC patients. **(B–E)** Higher expression of ACAA1, CAT, HMGCLL1, and PXMP4 indicated longer OS in LUAD patients. **(F–K)** Higher expression of NUDT19, PEX26, PEX5L, PEX6, SLC25A17, and SOD1 indicated shorter OS in LUAD patients. **(L)** The HRs of the high expression groups of the genes, in contrast to the low expression group. NSCLC, non-small cell lung cancer; LUSC, lung squamous carcinoma; LUAD, lung adenocarcinoma; C-DEPGs, common differentially expressed peroxisome pathway genes in NSCLC. Kaplan–Meier survival **(A–K)** and Cox regression analyses **(L)** were used and *p* < 0.05 was considered to be significant.

Through Kaplan-Meier survival analysis (Supplementary Table S2), among the 38 C-DEPGs, only HSD17B4 (Figure 2A) was shown to have prognostic effects on LUSC OS while 10 other genes (Figures 2B–K) were indicated to have significant prognostic effects on LUAD OS while no significance was shown for the other C-DEPGs (Supplementary Figures S1, S2). The 11 genes with significant prognostic effects were considered as K-DEPGs and their HRs were shown in Figure 2L. Notably, the down-regulated (ACAA1, CAT, HMGCCL1, and PXMP4) and up-regulated genes (NUCT19, PEX26, PEX5L, PEX6, SLC25A17, and SOD1) were shown to have favorable and unfavorable effects on LUAD OS, respectively. However, for HSD17B4, which was down-regulated in NSCLC, its unfavorable effects on LUSC OS were shown. It was indicated that although the genes were differentially expressed in both LUSC and LUAD, their prognostic effects were different in the two subtypes.

### Validation of the 11 K-DEPGs in Other NSCLC Datasets

**FIGURE 3:**
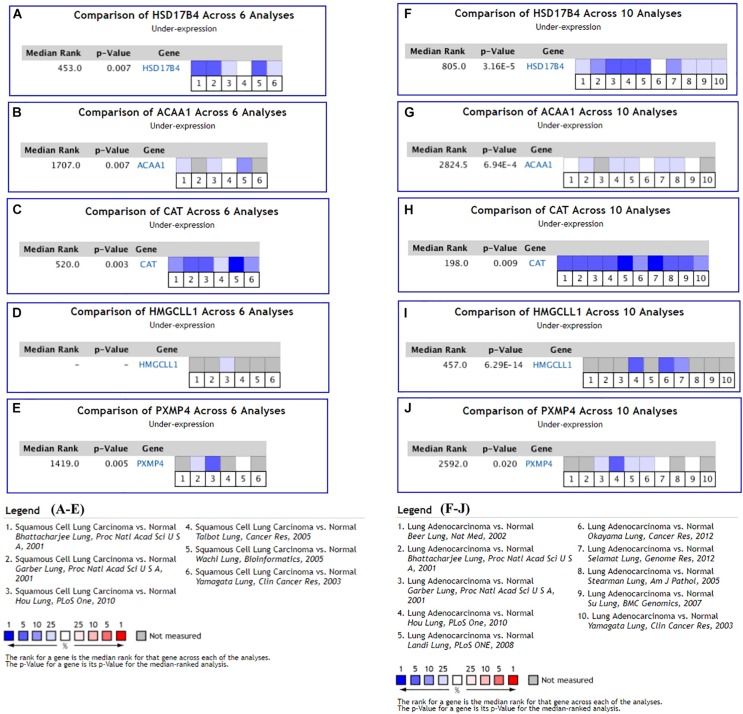
Oncomine meta-analyses of the down-regulated Key CDEPGs (K-DEPGs) in NSCLC. **(A–C)** Significant overall down-regulation of HSD17B4, ACAA1, and CAT in LUSC. **(D)** There only one LUSC dataset with the data of HMGCLL1 (Hou lung: fold change = –1.925, *p* < 0.01, Supplementary Table S1) and meta-analysis could not be performed. **(E)** PXMP4 was significantly down-regulated in LUSC. **(F–J)** significant overall down-regulation of HSD17B4, ACAA1, CAT, HMGCLL1, and PXMP4 in LUAD. NSCLC, non-small cell lung cancer; LUSC, lung squamous carcinoma; LUAD, lung adenocarcinoma; C-DEPGs, common differentially expressed peroxisome pathway genes. For all the analyses, *p* < 0.05 was considered significant.

**FIGURE 4:**
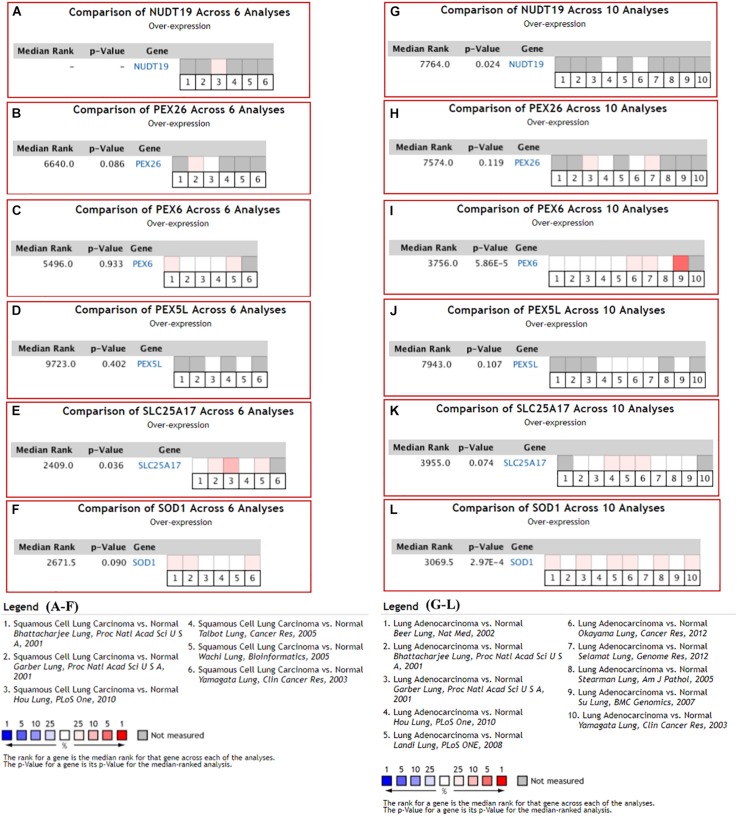
Oncomine meta-analysis of the up-regulated Key C-DEPGs (K-DEPGs) in NSCLC. **(A)** NUDT19 data was available only in Hou lung in LUSC (fold change = 1.535, *p* < 0.01, Supplementary Table S1) and there was not enough data for its meta-analysis. **(B–D,F)** there was no significant expressional difference of PEX26, PEX6, PEX5L, and SOD1 in LUSC. **(E)** SLC25A17 was significantly up-regulated in LUSC. **(G,I,L)** NUDT19, PEX6, and SOD1 was significantly up-regulated in LUAD. **(H,J)** PEX26 and PEX5Lwas not significantly up-regulated in LUAD. **(K)** There was a trend of up-regulation of SLC25A17 in LUAD. NSCLC, non-small cell lung cancer; LUSC, lung squamous carcinoma; LUAD, lung adenocarcinoma; C-DEPGs, common differentially expressed peroxisome pathway genes. For all the analyses, *p* < 0.05 was considered significant.

With the filters, 12 NSCLC datasets in Oncomine database were selected for expressional comparisons of the K-DEPGs between the NSCLC tumors and normal lung tissues. There were six and ten comparisons for LUSC and LUAD datasets, respectively. Except HMGCLL1 and NUDT19 in LUSC (Hou lung), there were two and above comparisons of the K-DEPGs each in LUSC or LUAD datasets (Supplementary Table S3) and meta analyses were applied to obtain the overall differences of the gene expressions. As shown in Figure 3, HSD17B4, ACAA1, CAT, HMGCLL1, and PXMP4 were all confirmed to be down-regulated in LUSC (Figures 3A–E) and LUAD (Figures 3F–J), respectively. Among the six up-regulated K-DEPGs, only NUDT19 were confirmed to be significantly increased in both LUSC (Figure 4A, Hou lung: fold change = 1.535, *p* < 0.01) and LUAD (Figure 4G) while no significance of PEX26 (Figures 4B,H, *p* > 0.05) and PEX5L (Figures 4D,J, *p* > 0.05) was shown in LUSC and LUAD. PEX6 (Figures 4C,I) and SOD1 (Figures 4F,L) were only confirmed to be up-regulated in LUAD while SLC25A17 (Figures 4E,K) was confirmed to be significantly increased in LUSC.

**FIGURE 5:**
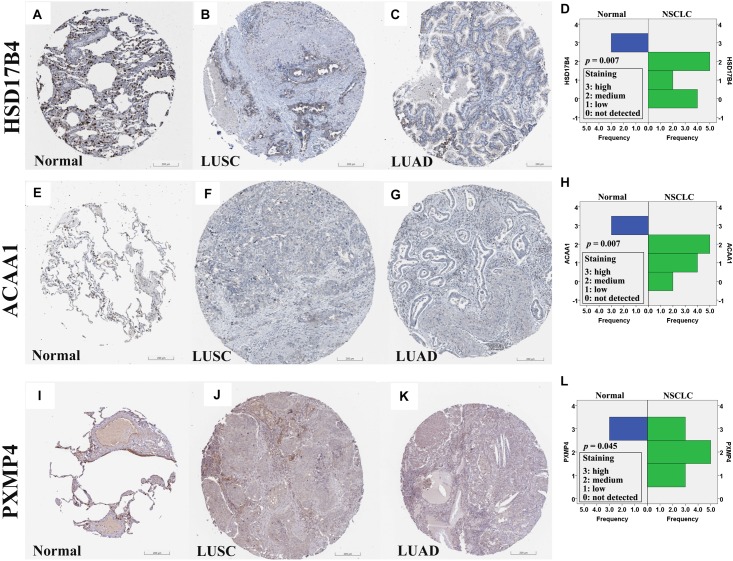
Immunohistochemical staining for HSD17B4, ACAA1, and PXMP4 in normal lung tissues and NSCLC tumors. **(A–C)** HSD17B4 staining in normal lung (high in pneumocytes), LUSC (low in tumor cells), and LUAD (low in tumor cells), respectively. **(E–G)** ACAA1 staining in normal lung (medium in pneumocytes), LUSC (low in tumor cells), and LUAD (low in tumor cells), respectively. **(I–K)** PXMP4 staining in normal lung (high in pneumocytes), LUSC (low in tumor cells), and LUAD (low in tumor cells), respectively. Through Wilcoxon test, HSD17B4 **(D)**, ACAA1 **(H)** and PXMP4 **(L)** was shown to be lower expressed in the NSCLC tumor cells than the normal lung pneumocytes, respectively. NSCLC, non-small cell lung cancer; LUSC, lung squamous carcinoma; LUAD, lung adenocarcinoma. All the immunostaining pictures were downloaded from HPA database. Antibody HPA021302 **(A–C)**, HPA007244 **(E–G)**, and CAB002427 **(I–K)** were used for staining.

**FIGURE 6:**
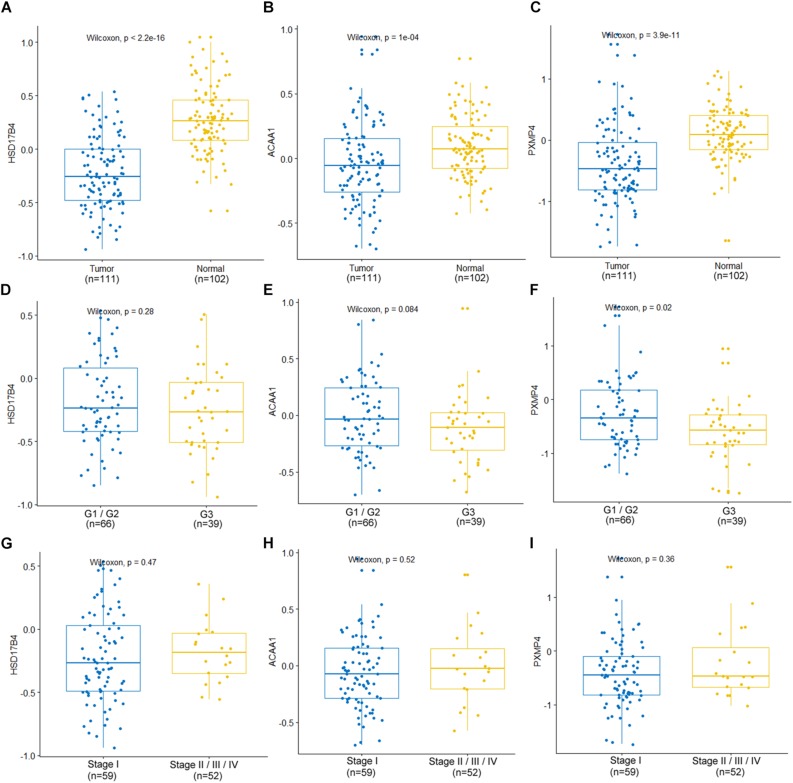
Expression profiles of HSD17B4, ACAA1, and PXMP4 in CPTAC lung cancer dataset. **(A–C)** Lower expression of HSD17B4, ACAA1 and PXMP4 in the tumors than the normal controls, respectively. **(D–F)** Expressional comparisons of HSD17B4, ACAA1 and PXMP4 between tumors with well-moderate differentiation (Grade 1 or Grade 2) and those with poor differentiation (Grade 3), respectively. **(G–I)** Expressional comparisons of HSD17B4, ACAA1, and PXMP4 between tumors of early stage (Stage I) and the late stage ones (Stage II or Stage III or Stage IV), respectively. The *y*-axis represented the protein abundance of the samples, with respect to the pooled reference sample, as log_2_ ratios. The *x*-axis represented different groups. CPTAC, Clinical Proteomic Tumor Analysis Consortium. Wilcoxon test was used and *p* < 0.05 was considered significant.

At protein level, for the six genes which were confirmed to be under-expressed (HSD17B4, ACAA1, CAT, HMGCLL1, and PXMP4) or over-expressed (NUDT19) both in LUSC and LUAD in above analyses, there was no IHC data available for HMGCLL1 and NUDT19 in HPA database and their expressional differences could not be obtained. For CAT, with negative staining both in lung cancer tumor cells and normal pneumocytes, no significant expressional difference was shown. However, for the other three genes, the IHC data indicated their down-regulation in NSCLC (Figure 5). HSD17B4, ACAA1, and PXMP4 were all strong expressed in lung (pneumocytes) while there was a negative to moderate expression of HSD17B4 and ACAA1 in the cytoplasm or membrane of LUSC and LUAD tumor cells. For PXMP4, although not all, most (9/12) of the NSCLC tumors also presented its negative to moderate expression and its decrease in the tumors was also obvious. In addition, as shown in Figure 6, the under-expression of HSD17B4 (Figure 6A), ACAA1 (Figure 6B), and PXMP4 (Figure 6C) was also shown in the CPTAC lung cancer dataset, consistent with their expression profiles in above analyses. Furthermore, through their comparisons between different grades (Figures 6D–F), a lower trend of ACAA1 expression (*p* = 0.084, 0.05 < *p* < 0.1) and a significant lower expression of PXMP4 (*p* = 0.02, *p* < 0.05) was shown in the poor differentiation tumors than the ones with well-moderate differentiation while no significant difference of HSD17B4 was shown. However, none of the three was shown to be statistically differentially expressed between the tumors between early and late stages (Figures 6G–I, *p* > 0.05).

### The Influences of Copy Number Variations and Methylation Values on the Expressions of HSD17B4, ACAA1, and PXMP4

**FIGURE 7:**
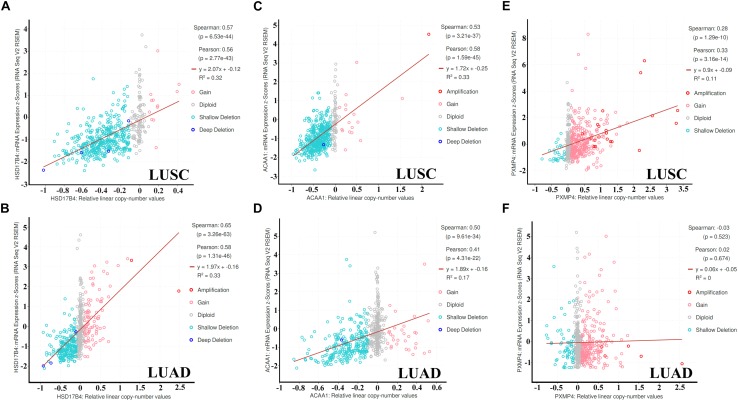
Correlations between HSD17B4, ACAA1, and PXMP4 expression and their copy number value in NSCLC. **(A,B)** Significant positive correlation between HSD17B4 CNV and HSD17B4 expression in LUSC and LUAD,respectively. **(C,D)** Significant positive correlation between ACAA1 CNV and ACAA1 expression in LUSC and LUAD,respectively. **(E)** Significant positive correlation between PXMP4 CNV and PXMP4 expression in LUSC. **(F)** There was no significant correlation between PXMP4 CNV and PXMP4 expression in LUAD. NSCLC, non-small cell lung cancer; LUSC, lung squamous carcinoma; LUAD, lung adenocarcinoma. Spearman and Pearson correlation analyses were used and *p* < 10^–5^ was considered significant.

As shown in Figure 7, through cBioPortal, HSD17B4 and ACAA1 expressions were shown to be positively correlated with their copy number values in both LUSC and LUAD. However, positive correlation between PXMP4 expression and its copy number value was observed in LUSC (Figure 7E) while not in LUAD (Figure 7F).

Through MEXPRESS analyses (Supplementary Table S4), interestingly, positive correlations were shown between HSD17B4 expression and its DNA methylation status of six CpG sites (cg23314948, cg06903010, cg24537512, cg16261704, cg01229506, and cg13432928) both in LUSC and LUAD. In contrast, significant negative correlations were shown between ACAA1 expression and its methylation value of CpG site cg10548708 in LUSC (Pearson *r* = −0.234, *p* = 4.412E-06) and LUAD (Pearson *r* = −0.411, *p* = 1.002E-18). For PXMP4, eight CpG sites (cg20588982, cg06231372, cg12297619, cg27361727, cg25092328, cg27194921, cg18669346, and cg24270031) in its promoter region were shown to have significant negative correlations between their methylation values and PXMP4 expression in the LUSC (Pearson *r* ranging from −0.374 to 0.457, *p* < 10^–5^) and LUAD (Pearson *r* ranging from −0.509 to 0.572, *p* < 10^–5^).

### NSCLC Specific Network of HSD17B4, ACAA1, and PXMP4 and Their Potential Functions in Immunoregulation and Tumor Growth Regulation in NSCLC

**FIGURE 8:**
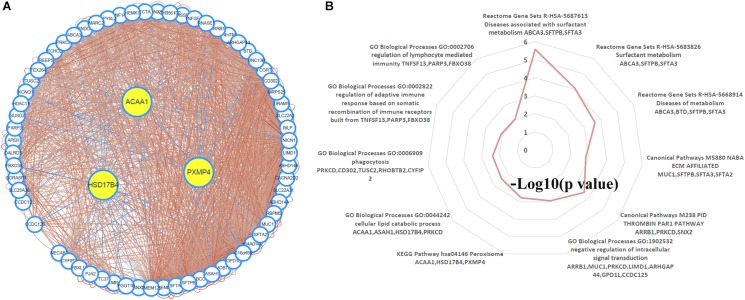
Construction and analysis of the NSCLC specific co-expression network of HSD17B4, ACAA1, and PXMP4. **(A)** NSCLC specific co-expression network of HSD17B4, ACAA1, and PXMP4. **(B)** Functional enrichments of the genes in the network. NSCLC, non-small cell lung cancer.

Through TCSBN and Cytoscape 3.6, the NSCLC co-expression network of HSD17B4, ACAA1, and PXMP4 was constructed (Figure 8A). There were 70 nodes and 1431 edges in the network and they represented the genes and their co-expression scores, respectively. The genes were applied to enrichment analysis and they were significant enriched in 11 terms (Figure 8B). Interestingly, except peroxisome, several immune-related processes including phagocytosis, regulation of adaptive immune response, regulation of lymphocyte mediated immunity, and ECM affiliated were enriched, consistent with the associations of peroxisomes with immune response and inflammation reported in previous studies (Vijayan et al., 2017; Di Cara et al., 2019), indicating the implication of HSD17B4, ACAA1, and PXMP4 in these processes.

**FIGURE 9:**
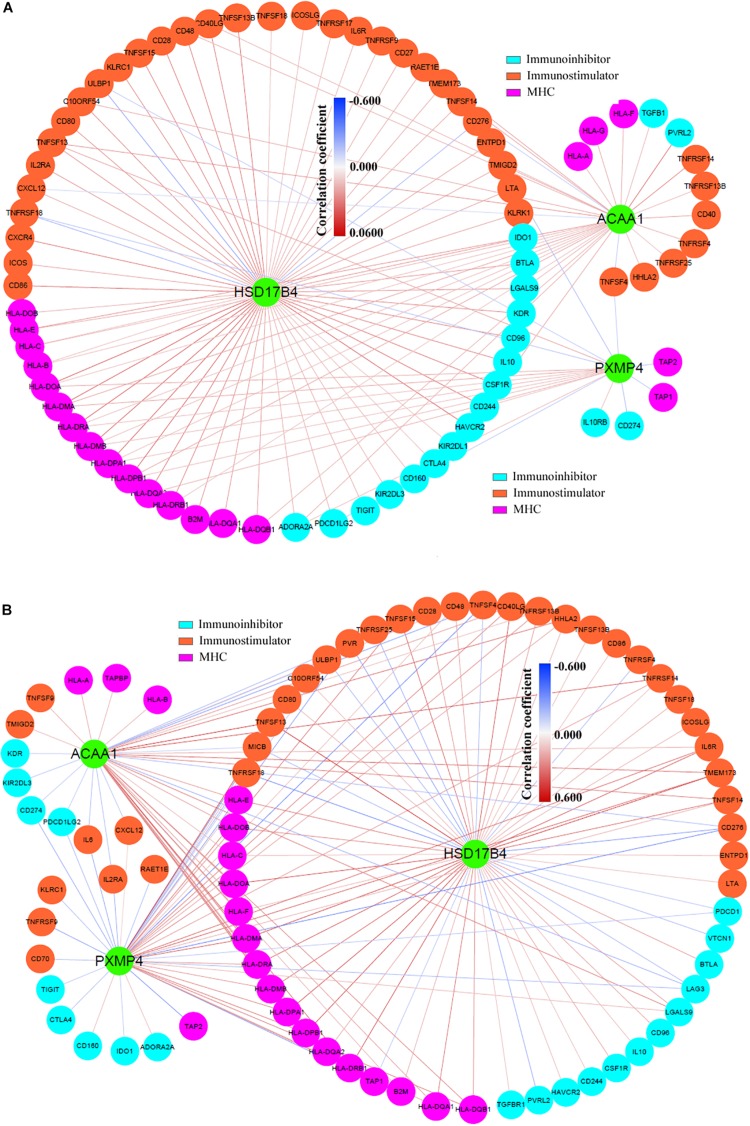
Significant **c**orrelations between HSD17B4, ACAA1, and PXMP4 expressions and immunomodulator gene expressions. **(A)** There were 114 significant positive or negative correlations between HSD17B4, ACAA1, and PXMP4 expressions and immunomodulator gene expressions in LUSC. **(B)** There were 145 significant positive or negative correlations between HSD17B4, ACAA1, and PXMP4 expressions and immunomodulator gene expressions in LUAD. The nodes and the edges represented the genes and the correlations, respectively. Purity-corrected Spearman correlation was used and *p* < 0.01 was considered significant.

In addition, via TIMER, the purity-corrected Spearman’s correlations between the expressions of HSD17B4, ACAA1, and PXMP4 and the immune infiltrations (Table 2) in NSCLC were obvious. Notably, HSD17B4 and ACAA1 were shown to be negatively correlated with LUSC purity while no significant correlation was found between the three gene expressions and LUAD purity (Table 2). However, both in LUSC and LUAD, HSD17B4 was found to have positive correlations with infiltrations of B cell, CD8^+^ T cell, Macrophage and dendritic cell. In contrast, ACAA1 was shown to be negatively correlated with CD8^+^ T cell and neutrophil infiltrations only in LUAD but not in LUSC. For PXMP4, its positive correlations with macrophage in LUSC while its significant correlations with B cell and neutrophil in LUAD were indicated. Consistent with their correlations with the immune infiltrations, the three gene expressions were shown to be significantly correlated with most (in LUSC: 77/91, in LUAD: 77/91, in both LUSC and LUAD: 67/91) of the immunomodulator gene expressions with 116 and 145 significant positive/negative correlations in LUSC (Figure 9A) and LUAD (Figure 9B), respectively. Although there were more positive correlations than the negative ones, the three gene expressions were positively or negatively correlated with both immunostimulatory and the immunoinhibitory gene expressions, indicating their potential roles in the immune inhibition and/or immune stimulation of NSCLC. Interestingly, most of the significant correlations (LUSC: 36/38, LUAD: 44/47) between the three gene expressions and MHC-related gene expressions were positive ones. Considering the crucial roles of MHC molecules in presenting antigens to lymphocytes and that (Lenz, 2018), the positive roles of HSD17B4, ACAA1, and PXMP4 in regulation of antigen presentation were also indicated. In addition, among the top 10 correlations (with the lowest *p*-values) (Supplementary Table S5), all the 10 correlation in LUSC and 7 of 10 correlations in LUAD were associated with HSD17B4 and all the correlations were positive ones, indicating although all the three genes were associated immune response, the immunoregulatory potential of HSD17B4 in NSCLC was highlighted.

**TABLE 2:** The correlations between HSD17B4, ACAA1, and PXMP4 expression and immune infiltrations in NSCLC.

		LUSC		LUAD	
	Variable	Partial cor.	*p*-value	Partial cor.	*p*-value
HSD17B4	Purity	–0.303	1.416E-11**	–0.024	0.598
	B Cell	0.214	2.854E-06**	0.220	1.021E-06**
	CD8^+^ T cell	0.242	9.521E-08**	0.092	0.043*
	CD4^+^ T cell	0.0478	0.299	0.048	0.296
	Macrophage	0.267	3.139E-09**	0.168	1.975E-04**
	Neutrophil	0.243	8.123E-08**	0.082	0.073
	Dendritic cell	0.253	2.248E-08**	0.187	3.341E-05**

ACAA1	Purity	–0.236	1.758E-07**	–0.057	0.206
	B Cell	–0.024	0.600	0.034	0.453
	CD8^+^ T cell	–0.027	0.556	–0.147	0.001**
	CD4^+^ T cell	0.099	0.030*	0.028	0.532
	Macrophage	0.036	0.428	–0.005	0.918
	Neutrophil	–0.030	0.516	–0.142	0.002**
	Dendritic cell	0.053	0.253	–0.042	0.353

PXMP4	Purity	–0.023	0.624	0.082	0.069
	B Cell	0.045	0.324	0.192	2.204E-05**
	CD8^+^ T Cell	0.039	0.402	–0.020	0.657
	CD4^+^ T Cell	0.025	0.586	0.045	0.320
	Macrophage	0.254	1.744E-08**	0.077	0.089
	Neutrophil	–0.030	0.514	–0.120	0.009**
	Dendritic cell	0.049	0.291	0.024	0.590

NSCLC, non-small cell lung cancer; LUSC, lung squamous carcinoma; LUAD, lung adenocarcinoma. *p < 0.05; **p < 0.01. Spearman’s purity-corrected correlation analysis was used for and p < 0.01 was considered significant. Partial cor., partial correlation.

Through TIMER, higher MKI67 expression was shown to be in LUSC and LUAD tumors than their normal controls (Supplementary Figures S3A,B, *p* < 0.01). And MKI67 expression was negatively correlated with expressions of HSD17B4, ACAA1, and PXMP4 in both LUSC and LUAD (Supplementary Figures S3C,D, *p* < 0.01). At protein level, positive expression of MKI67 in NSCLC tumors while negative expressed in normal lung tissues was shown in HPA (Supplementary Figures S3E–G) and MKI67 overexpression in NSCLC tumors was also confirmed in CPTAC lung cancer dataset (Supplementary Figure S3H, *p* < 0.01). Although no significant correlation between HSD17B4 and MIKI67 expression (Supplementary Figure S3I, *p* > 0.05) was shown in CPTAC lung cancer, the other two proteins (ACAA1 and PXMP4) were confirmed to be negatively correlated with MKI67 expression (Supplementary Figures S3J,K, *p* < 0.01), indicating their potential roles in regulation of NSCLC growth.

### HSD17B4, ACAA1, and PXMP4 Expressions and Anti-cancer Drug Sensitivity in NSCLC

**FIGURE 10:**
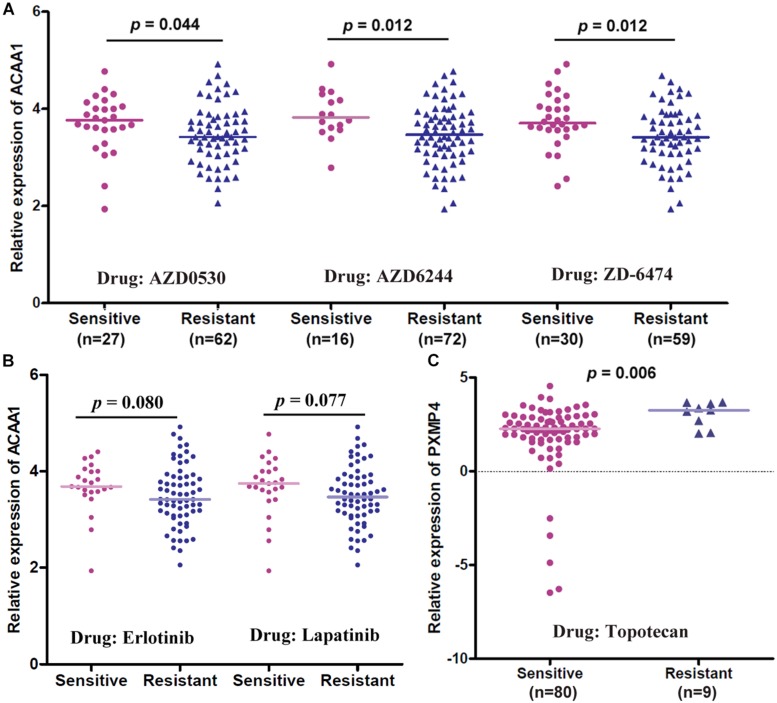
Gene expressional differences of ACAA1 and PXMP4 between NSCLC cell lines with different anti-cancer drug sensitivities. **(A)** For anti-cancer drugs AZD0530, AZD6244, and ZD6474, ACAA1 was higher expressed in drug-sensitive NSCLC cell lines than the drug-resistant ones. **(B)** For Erlotinib and Lapatinib, there was a higher trend (0.05 < *p* < 0.1) of ACAA1 expression in the sensitive cell lines than the resistant ones. **(C)** PXMP4 was higher expressed in the Topotecan-sensitive NSCLC cell lines than the resistant ones. NSCLC, non-small cell lung cancer. Two independent samples Mann–Whitney *U* test was used for the comparisons and *p* < 0.05 was considered significant.

Through correlation analyses (Supplementary Table S6), ACAA1 expression was shown to be negatively correlated with the IC50 values of five anti-cancer drugs including AZD0530 (*r* = −0.261, *p* = 0.014), AZD6244 (*r* = −0.281, *p* = 0.008), Erlotinib (*r* = −0.211, *p* = 0.047), Lapatinib (*r* = −0.234, *p* = 0.028), and ZD-6474 (*r* = −0.285, *p* = 0.007) while no significant correlation was shown for HSD17B4 or PXMP4 expression with the IC50 values of all the 24 drugs (*p* > 0.05). The expressional differences of the three genes between the sensitive cell lines and the resistant ones were shown in Table 3, consistent with the negative correlations above, for anti-cancer drugs AZD0530, AZD6244, and ZD-6474, there was a significant higher expression of ACAA1 in the sensitive cell lines than in the resistant ones (Figure 10A). For Erlotinib and Lapatinib, a higher trend of ACAA1 expression in the sensitive cell lines were shown (Figure 10B, 0.1 > *p* > 0.05). Interestingly, although no significant correlation was shown between PXMP4 expression and Topotecan IC50 values, there was a significant lower expression of PXMP4 in the Topotecan-sensitive NSCLC cell lines than the resistant ones (Figure 10C), indicating that the expressional difference of PXMP4 might be due to the differences between the two groups but not due to the drug itself.

**TABLE 3:** Expressional differences of HSD17B4, ACAA1, and PXMP4 between NSCLC cell lines with different anti-cancer drug sensitivity.

Compound	Target	Sensitive (*n*)	Resistant (*n*)	HSD17B4 (*p*-value)	ACAA1 (*p*-value)	PXMP4 (*p*-value)
17-AAG	HSP90	79	10	0.071	0.122	0.232
AEW541	IGF1R	39	50	0.667	0.704	0.342
AZD0530	ABL	27	62	0.120	0.044*	0.901
AZD6244	MEK	16	72	0.545	0.012*	0.787
Erlotinib	EGFR	23	66	0.687	0.080	0.442
Irinotecan^†^	TOP1	45	0	NA	NA	NA
L-685458	GC	13	73	0.146	0.957	0.409
Lapatinib	EGFR	25	64	0.834	0.077	0.294
LBW242	XIAP	9	79	0.495	0.175	0.374
Nilotinib	ABL	16	56	0.074	0.735	0.626
Nutlin-3	MDM2	1	88	0.139	0.436	0.276
Paclitaxel	TUBB1	80	9	0.406	0.785	0.391
Panobinostat^†^	HDAC	87	0	NA	NA	NA
PD-0325901	MEK	36	53	0.581	0.135	0.245
PD-0332991	CDK4	5	67	0.938	0.603	0.528
PF2341066	c-MET	20	69	0.169	0.523	0.898
PHA-665752	c-MET	3	85	0.991	0.498	0.827
PLX4720	RAF	10	79	0.269	0.533	0.559
RAF265	RAF	52	26	0.824	0.433	0.266
Sorafenib	RTK	16	73	0.593	0.677	0.423
TAE684	ALK	61	28	0.874	0.485	0.902
TKI258	FGFR	36	53	0.719	0.694	0.94
Topotecan	TOP1	80	9	0.522	0.849	0.006**
ZD-6474	EGFR	30	59	0.466	0.012*	0.639

*p < 0.05; **p < 0.01; ^†^No resistant NSCLC cell line was shown for the drugs and expressional comparisons were not available. For the other 22 drugs, Mann–Whitney U test was used for the gene expression comparisons between the sensitive NSCLC cell lines and the resistant ones. NSCLC, non-small cell lung cancer. NA, not available. For all the analyses, p < 0.05 was considered significant.

## Discussion

Reprogramming of metabolic pathways are implicated in the process of tumor development to facilize its unregulated growth and metastatic dissemination (Hanahan and Weinberg, 2011). Recently, the involvement of peroxisome pathway in cancer was demonstrated (Dahabieh et al., 2018). However, although dysfunctions of peroxisomes were shown in many tumors (De Craemer, 1995; Valenca et al., 2015), their roles in lung cancer were rarely explored. Here, we focused on the peroxisomes in NSCLC and identified 38 C-DEPGs in LUSC and LUAD. Among the 38 genes, HSD17B4, ACAA1, and PXMP4 were highlighted for their down-regulation both at mRNA level and protein level, their prognostic effects, their correlations with immune infiltrations, immunomodulator gene expressions, the proliferation marker (MKI67) expression and/or anti-cancer drug sensitivities in NSCLC.

HSD17B4 encodes 17β-hydroxysteroid dehydrogenase type 4 (HSD17B4), a 80 kDa multifunctional enzyme localized in peroxisomes, also known as D-specific bifunctional protein (DBP) and multifunctional protein 2 (MFP-2)(de Launoit and Adamski, 1999). In normal cells, HSD17B4 plays important roles in the peroxisomal β-oxidation of long- and branched-chain fatty acids (Baes et al., 2000; Breitling et al., 2001), bile acid biosynthesis (Ferdinandusse et al., 2005), and sterol metabolism (Poutanen et al., 1995). Over the last decade, HSD17B4 has been reported to be involved in the tumorigenesis and progression of many tumors. However, the specific roles of HSD17B4 seems to be inconsistent in different contexts. For example, its overexpression was associated with the tumor cell proliferation in liver cancer (Lu et al., 2015, 2019), prostate cancer and colon cancer (Frasinyuk et al., 2017) while exerted tumor suppressive functions in adrenocortical carcinoma (Ding et al., 2017). Here, HSD17B4 was shown to be under-expressed in NSCLC, indicating its diagnostic potential. Consistent with its positive correlation with inflammation in liver cancer tissues (Pan et al., 2018), here, its significant correlations with the immune cell infiltrations and immunomodulator gene expressions were obvious in NSCLC, indicating the important roles of HSD17B4 in immune response regulation. Considering the important roles of immune dysfunction during lung cancer occurrence and progression (Datar and Schalper, 2016; Mascaux et al., 2019), the potential effects of HSD17B4 in immunoregulation might provide new clues for NSCLC immunotherapy. However, its unfavorable prognostic effects were shown in LUSC while not in LUAD, indicating the heterogeneity of its roles in different lung cancer subtypes. Further study is needed for investigation of its specific activities in NSCLC.

ACAA1 gene encodes acetyl-CoA acyltransferase 1 (ACAA1), another important enzyme during peroxisomal β-oxidation of fatty acids (Antonenkov et al., 1997). In previous studies, it was demonstrated to be under-expressed in liver cancer (Yan et al., 2017) and kidney renal clear cell carcinoma (Zhang et al., 2019). Here, similar to HSD17B4, ACAA1 was also found to be down-regulated and negatively correlated with MKI67 expression in NSCLC, indicating its anti-tumor potential. Notably, considering the importance of HSD17B4 and ACAA1 in peroxisomal β-oxidation of very long fatty acids, the dysfunction of the process in NSCLC was indicated, consistent with the anti-tumor function of β-oxidation in lung cancer in a previous study (Srivastava et al., 2014). In addition, as one of the innate immunity genes, the single nucleotide polymorphisms (SNPs) of ACAA1 have been reported to be associated with pathogenesis of childhood asthma (Sharma et al., 2012) and the protective effects of its exposure to endotoxin (Sordillo et al., 2011). In this study, interestingly, besides its negative correlations with CD8^+^ T cell and neutrophil infiltrations in LUAD, its significant correlations with immunomodulator gene expressions was presented in both LUSC and LUAD, indicating its associations with innate and adaptive immune response. Furthermore, lower ACAA1 expression was shown to be associated the resistance of NSCLC cell lines to five different anti-cancer drugs including AZD0530, AZD6244, ZD-6474, Erlotinib and Lapatinib, providing new clues for NSCLC chemotherapy.

PXMP4, also termed PMP24, a 24-kDa peroxisomal integral membrane protein, was first isolated from rat liver peroxisome membranes (Reguenga et al., 1999). Although it was known to be able to bind chaperone/membrane transporter PEX19 (Sacksteder et al., 2000), its function in peroxisomes was unclear. However, its dysfunction in prostate cancer was reported and its under-expression was associated with the DNA hypermethylation of its CpG island (Wu and Ho, 2004; Zhang et al., 2010). Here, we also found the down-regulation of PXMP4 and the negative correlations between its expression and the methylation values of its CpG sites in LUSC and LUAD. However, PXMP4 expression was shown to be positively correlated with its copy numbers in LUSC while not in LUAD, indicating the difference between the differences of PXMP4 regulation between the two subtypes. Furthermore, its negative correlation with MKI67 expression and LUAD differentiation indicated the tumor-suppressor activities. In addition, besides their prognostic effects in LUSC or LUAD, since there was no expressional difference of HSD17B4, ACAA1, and PXMP4 between NSCLC tumors of early and late stages, their prognostic and diagnostic potential was shown.

In summary, dy-regulation of peroxisome pathway was common in NSCLC and more than 30% of peroxisome pathway genes were consistently down- or up-regulated in LUSC and LUAD, indicating their crucial roles in NSCLC. To our knowledge, this is the first study for the systemic investigation of peroxisomes in lung cancer. The C-DEPGs might provide new clues for the study of peroxisomes in NSCLC. HSD17B4, ACAA1, and PXMP4 might be new markers for NSCLC diagnosis and prognosis and/or new therapeutic targets for NSCLC treatment. In addition, HSD17B4 and ACAA1 down-regulation highlighted the potential effects of dysfunction of peroxisomal β-oxidation of fatty acids in NSCLC. However, considering the differences in their prognostic effects, regulations, correlations with tumor purity and immune infiltrations effects between the two subtypes, further study is needed to investigate the specific functions of HSD17B4, ACAA1, and PXMP4 in LUSC and LUAD, respectively.

## Data Availability Statement

The data that support the findings of this study are available in TCGA (http://cancergenome.nih.gov), Oncomine (http://www.oncomine.org), cBioPortal (https://www.cbioportal.org/), and TIMER (https://cistrome.shinyapps.io/timer/).

## Author Contributions

XZ and LD conceived and designed the study. XZ, HY, and FG collected and analyzed the data. XZ and JZ interpreted the data. XZ and HY drafted the manuscript. DL reviewed and revised the manuscript. All authors read and approved the final manuscript.

## Conflict of Interest

The authors declare that the research was conducted in the absence of any commercial or financial relationships that could be construed as a potential conflict of interest.

## Footnotes

1 https://portal.gdc.cancer.gov/

2 https://www.kegg.jp/

3 http://gepia.cancer-pku.cn/

4 https://cptac-data-portal.georgetown.edu/

5 http://www.cbioportal.org/

6 http://mexpress.be

7 http://inetmodels.com/

8 http://metascape.org/

9 https://cistrome.shinyapps.io/timer/

10 https://tcia.at/

11 https://portals.broadinstitute.org/ccle/
