# Supplementary material for: HSD17B4, ACAA1, and PXMP4 in Peroxisome Pathway Are Down-Regulated and Have Clinical Significance in Non-small Cell Lung Cancer
Source: Front Genet. 2020 Mar 20;11:273. doi: 10.3389/fgene.2020.00273 (PMC7103649; doi:10.3389/fgene.2020.00273)
Supplement: Supplementary file 1 [file Data_Sheet_1.PDF]

Supplementary Material

1 Supplementary Tables

Supplementary Table S1. Clinical features of CPTAC lung cancer patients.

Clinical features	Overall (n=111)
Tumor type	Lung adenocarcinoma
Age (year)	n (%)
≤ 60	48 (43.2%)
> 60	63 (57.8%)
Sex	n (%)
Male	73 (65.8%)
Female	38 (34.2%)
TNM stage	n (%)
Stage I	59 (53.2%)
Stage II	30 (27.0%)
Stage III	21(18.9%)
Stage IV	1 (0.9%)
Grade	n (%)
G1	7(6.3%)
G2	59(53.2%)
G3	39(35.1%)
NA	6 (5.4%)

CPTAC, Clinical Proteomic Tumor Analysis Consortium; NA, not available.

Supplementary Table S2. Kaplan-Meier survival analyses of the C-DEPGs in NSCLC

Gene	LUSC (log rank *p* value)	LUAD (log rank *p* value)
ACAA1	0.251	0.006**
ACSL1	0.917	0.935
ACSL4	0.312	0.054
AGXT	0.119	0.884
AMACR	0.438	0.464
CAT	0.500	0.003**
DAO	0.938	0.111
DDO	0.082	0.179
DECR2	0.917	0.907
EPHX2	0.860	0.108
FAR2	0.223	0.436
GNPAT	0.941	0.161
HACL1	0.263	0.057
HMGCLL1	0.390	0.009**
HSD17B4	0.022*	0.436
IDH1	0.767	0.472
IDH2	0.081	0.396
MLYCD	0.956	0.175
MPV17	0.925	0.245
MPV17L2	0.399	0.868
NUDT19	0.736	0.024*
PECR	0.838	0.707
PEX1	0.109	0.649
PEX10	0.185	0.773
PEX11G	0.528	0.594
PEX13	0.950	0.754
PEX16	0.654	0.715
PEX26	0.555	0.017*
PEX5L	0.992	0.015*
PEX6	0.435	0.044*
PIPOX	0.955	0.105
PRDX1	0.604	0.374
PXMP2	0.708	0.256
PXMP4	0.800	4.773E-04**
SLC25A17	0.158	0.020*
SOD1	0.223	0.001**
SOD2	0.392	0.198
XDH	0.093	0.098

*, $p<0.05$; **, $p<0.01$. Kaplan-Meier survival analysis with log rank test was used for survival analyses and $p<0.05$ was considered statistically significant.

Supplementary Table S3. Expressional differences of the K-DEPGs in NSCLC datasets from Oncomine database

Comparison	HSD17B4	ACAA1	CAT	HMGCLL1	PXMP4
Talbot lung (n=93)					
LUSC vs. normal	FC=-1.003<br>P=0.486	FC=1.018<br>P=0.664	FC=-1.589<br>P=3.35E-4	NA	NA
Garber Lung (n=73)					
LUAD vs. normal	FC=-1.592<br>P=2.09E-4	NA	FC=-1.394<br>P=2.79E-5	NA	FC=-1.506<br>P=0.037
LUSC vs. normal	FC=-2.689<br>P=4.29E-7	NA	FC=-2.403<br>P=2.62E-5	NA	FC=-2.001<br>P=0.005
Hou lung (n=156)					
LUAD vs. normal	FC=-1.717<br>P=2.22E-12	FC=-1.313<br>P=2.52E-5	FC=-2.439<br>P=3.91E-18	FC=-1.869<br>P=4.71E-16	FC=-1.727<br>P=3.00E-14
LUSC vs. normal	FC=-2.154<br>P=3.37E-10	FC=-1.347<br>P=1.22E-8	FC=-3.427<br>P=6.33E-15	FC=-1.925<br>P=3.73E-9	FC=1.975<br>P=1.07E-16
Bhattacharjee lung(n=203)					
LUAD vs. normal	FC=-3.602<br>P=6.65E-5	FC=-2.385<br>P=0.003	FC=-4.860<br>P=2.00E-5	NA	NA
LUSC vs. normal	FC=-6.685<br>P=1.42E-6	FC=-2.104<br>P=0.014	FC=-2.579<br>P=0.006	NA	NA
Stearman Lung (n=39)					
LUAD vs. normal	FC=-1.199<br>P=0.043	FC=-1.218<br>P=7.35E-4	FC=-2.459<br>P=1.51E-8	NA	NA
Wachi lung (n=10)					
LUSC vs. normal	FC=-3.106<br>P=9.99E-4	FC=-1.840<br>P=0.002	FC=-3.582<br>P=2.75E-5	NA	FC=-1.161<br>P=0.116
Su lung (n=66)					
LUAD vs. normal	FC=-1.428<br>P=4.49E-4	FC=-1.148<br>P=0.044	FC=-2.645<br>P=1.14E-9	NA	FC=1.280<br>P=0.003
Okayama lung (n=246)					
LUAD vs. normal	FC=-1.082<br>P=0.043	FC=1.146<br>P=0.013	FC=-1.622<br>P=1.42E-9	FC=-2.670<br>P=6.29E-14	FC=-1.579<br>P=6.73E-7
Landi Lung (n=107)					
LUAD vs. normal	FC=-1.653<br>P=7.29E-15	FC=-1.206<br>P=0.001	FC=-2.677<br>P=2.40E-31	NA	FC=-1.118<br>P=0.003
Selamat Lung (n=116)					
LUAD vs. normal	FC=-1.485<br>P=1.31E-9	FC=-1.297<br>P=4.98E-6	FC=-3.566<br>P=1.24E-29	FC=-1.154<br>P=4.69E-11	FC=-1.160<br>P=0.018
Beer Lung (n=96)					
LUAD vs. normal	FC=-1.559<br>P=6.32E-5	FC=-1.146<br>P=0.041	FC=-1.476<br>P=1.05E-8	NA	NA
Yamagata Lung (n=31)					
LUAD vs. normal	FC=-1.494<br>P=0.043	NA	FC=-1.439<br>P=0.018	NA	NA
LUSC vs. normal	FC=-1.826<br>P=0.012	NA	FC=-1.849<br>P=0.004	NA	NA

Supplementary Table S3 - continued

Comparison	NUDT19	PEX26	PEX5L	PEX6	SLC25A17	SOD1
Talbot lung (n=93)						
LUSC vs. normal	NA	NA	NA	FC=-1.103<br>P=0.067	FC=-1.021<br>P=0.288	FC=1.115<br>P=0.173
Garber Lung (n=73)						
LUAD vs. normal	NA	FC=1.281<br>P=0.017	NA	FC=1.370<br>P=0.111	FC=1.340<br>P=0.042	FC=1.395<br>P=0.021
LUSC vs. normal	NA	FC=1.811<br>P=0.017	NA	FC=-1.129<br>P=0.334	FC=1.379<br>P=0.036	FC=1.534<br>P=0.010
Hou lung (n=156)						
LUAD vs. normal	FC=1.201<br>P=0.002	FC=1.016<br>P=0.155	FC=1.017<br>P=0.175	FC=1.277<br>P=0.002	FC=1.270<br>P=7.81E-6	FC=1.140<br>P=0.002
LUSC vs. normal	FC=1.535<br>P=1.51E-4	FC=1.035<br>P=0.155	FC=-1.004<br>P=0.440	FC=1.157<br>P=0.122	FC=1.377<br>P=3.99E-8	FC=1.258<br>P=6.21E-4
Bhattacharjee lung(n=203)						
LUAD vs. normal	NA	NA	NA	FC=1.031<br>P=0.446	FC=-2.131<br>P=0.006	FC=1.628<br>P=0.111
LUSC vs. normal	NA	NA	NA	FC=1.516<br>P=0.446	FC=1.116<br>P=0.377	FC=-1.796<br>P=0.032
Stearman Lung (n=39)						
LUAD vs. normal	NA	NA	NA	FC=1.092<br>P=0.239	FC=1.017<br>P=0.474	FC=1.266<br>P=8.67E-4
Wachi lung (n=10)						
LUSC vs. normal	NA	NA	FC=1.036<br>P=0.244	FC=1.340<br>P=0.050	FC=1.133<br>P=0.014	FC=1.444<br>P=0.070
Su lung (n=66)						
LUAD vs. normal	NA	FC=-1.110<br>P=0.066	FC=-1.014<br>P=0.475	FC=3.337<br>P=9.94E-6	FC=1.115<br>P=0.107	FC=1.184<br>P=0.044
Okayama lung (n=246)						
LUAD vs. normal	FC=1.082<br>P=0.046	NA	FC=1.105<br>P=0.314	FC=1.615<br>P=1.37E-4	FC=1.303<br>P=1.36E-7	FC=1.223<br>P=8.97E-7
Landi Lung (n=107)						
LUAD vs. normal	NA	NA	FC=1.002<br>P=0.478	FC=1.231<br>P=6.55E-4	FC=1.289<br>P=8.42E-7	FC=1.193<br>P=5.93E-4
Selamat Lung (n=116)						
LUAD vs. normal	NA	FC=1.149<br>P=5.65E-7	FC=1.018<br>P=0.107	FC=1.392<br>P=5.56E-5	FC=-1.012<br>P=0.334	FC=1.164<br>P=0.006
Beer Lung (n=96)						
LUAD vs. normal	NA	NA	NA	FC=1.171<br>P=0.094	NA	FC=1.154<br>P=0.006
Yamagata Lung (n=31)						
LUAD vs. normal	NA	NA	NA	NA	NA	FC=1.342<br>P=0.072
LUSC vs. normal	NA	NA	NA	NA	NA	FC=1.558<br>P=0.012

FC, fold change; NA, not available. Two independent samples T test was used and $p < 0.05$ was considered significant. K-DEPGs, key common differentially expressed peroxisome pathway genes in LUSC and LUAD. NSCLC, non-small cell lung cancer; LUSC, lung squamous carcinoma; LUAD, lung adenocarcinoma.

Supplementary Table S4. Correlations between the expressions of HSD17B4, ACAA1 and PXMP4 and their methylation values

		LUSC		LUAD	
	CpG site	Pearson *r*	*p* value	Pearson *r*	*p* value
HSD17B4	cg23314948	0.332	6.719E-11**	0.400	5.864E-18**
	cg06903010	0.319	3.388E-10**	0.272	2.101E-09**
	cg24537512	0.309	1.251E-09**	0.377	2.788E-16**
	cg16261704	0.300	3.669E-09**	0.277	9.968E-10**
	cg01229506	0.290	1.158E-08**	0.291	1.399E-10**
	cg13432928	0.288	1.483E-08**	0.377	2.698E-16**
	cg20964589	-0.274	7.335E-08**	-0.175	1.199E-04
	cg21035905	0.256	4.779E-07**	0.286	2.840E-10**
	cg05185946	0.244	1.809E-06**	0.114	0.013
	cg11398794	0.236	3.558E-06**	0.343	6.398E-14**
	cg09486093	0.232	5.352E-06**	-0.112	0.014
	cg21878514	0.220	1.589E-05	-0.189	3.298E-05
	cg11567627	0.217	2.237E-05	0.409	1.309E-18**
	cg08929903	0.215	2.550E-05	0.326	8.359E-13**
	cg10647644	-0.173	7.528E-04	-0.024	0.604
	cg17871537	0.167	0.001	0.159	4.956E-04
	cg26974158	0.161	0.002	0.112	0.015
	cg17593721	0.156	0.002	-0.123	0.007
	cg22067069	0.149	0.004	0.028	0.547
	cg18399472	0.127	0.014	0.312	7.213E-12**
	cg11532800	-0.119	0.021	-0.009	0.850
	cg15896301	0.105	0.042	0.091	0.047
	cg14874121	-0.099	0.056	0.049	0.282
	cg13600362	-0.089	0.084	0.016	0.722
	cg12156838	0.032	0.530	-0.251	3.350E-08**
	cg04473621	-0.031	0.548	-0.134	0.003
	cg13716421	-0.023	0.661	-0.045	0.328
	cg15028082	NA	NA	NA	NA
	cg11027879	NA	NA	NA	NA
	cg24663638	NA	NA	NA	NA
	cg10959673	NA	NA	NA	NA
	cg18775566	NA	NA	NA	NA
	ch.5.2173511R	NA	NA	NA	NA
	cg07029084	NA	NA	NA	NA
	cg24561266	NA	NA	NA	NA
ACAA1	cg00884680	-0.246	1.388E-06**	-0.085	0.063
	cg10548708	-0.234	4.412E-06**	-0.411	1.002E-18**

	cg02314846	-0.180	4.314E-04	-0.042	0.358
	cg17696234	-0.173	7.251E-04	0.024	0.608
	cg17877494	-0.149	0.004	0.013	0.769
	cg27043630	-0.127	0.014	-0.038	0.410
	cg08548888	-0.127	0.014	-0.110	0.016
	cg15348640	-0.120	0.020	0.004	0.935
	cg17034030	-0.103	0.045	0.040	0.390
	cg06239037	-0.100	0.052	-0.046	0.318
	cg24368167	-0.095	0.066	0.030	0.518
	cg15107884	-0.092	0.075	-0.024	0.608
	cg01702246	0.080	0.122	0.052	0.258
	cg06069310	-0.079	0.127	0.197	1.504E-05
	cg06808967	-0.073	0.155	0.021	0.641
	cg15427004	-0.064	0.217	0.062	0.179
	cg02829783	-0.061	0.238	0.087	0.059
	cg01353464	-0.052	0.318	-0.063	0.170
	cg07895684	-0.047	0.368	0.125	0.006
	cg25525163	-0.024	0.646	-0.160	4.641E-04
	cg00343022	-0.007	0.900	-0.048	0.296
	cg00091063	NA	NA	NA	NA
PXMP4	cg20588982	-0.457	9.750E-19**	-0.572	1.746E-30**
	cg06231372	-0.405	2.396E-15**	-0.570	2.520E-30**
	cg12297619	-0.419	3.231E-16**	-0.570	2.762E-30**
	cg27361727	-0.395	9.815E-15**	-0.551	6.010E-29**
	cg25092328	-0.400	5.038E-15**	-0.546	1.419E-28**
	cg27194921	-0.399	5.516E-15**	-0.542	2.639E-28**
	cg18669346	-0.380	8.447E-14**	-0.523	5.992E-27**
	cg24270031	-0.374	1.997E-13**	-0.509	6.538E-26**
	cg15651928	-0.031	0.552	-0.350	1.955E-14**
	cg04730850	-0.105	0.041	-0.172	1.569E-04
	cg04988273	-0.118	0.022	-0.155	6.949E-04
	cg26537568	NA	NA	NA	NA
	cg01260366	NA	NA	NA	NA
	cg25358761	NA	NA	NA	NA

NA, not available; **, $p < 10^{-5}$. Pearson correlation analysis was used and $p < 10^{-5}$ was considered statistically significant.

Supplementary Table S5. The top 10 correlations of HSD17B4, ACAA1 and PXMP4 with immunomodulator genes in NSCLC.

NSCLC	Peroxisomal gene	Immunomodulatory gene		Partial.cor	*P* value
		Gene symbol	Type		
LUSC	HSD17B4	CXCR4	Immunostimulator	0.309	1.369E-22
	HSD17B4	HLA-DOA	MHC	0.374	2.737E-17
	HSD17B4	HLA-DMA	MHC	0.364	2.026E-16
	HSD17B4	HLA-DRA	MHC	0.362	3.084E-16
	HSD17B4	HLA-DMB	MHC	0.358	7.597E-16
	HSD17B4	HLA-DPA1	MHC	0.352	2.124E-15
	HSD17B4	HAVCR2	Immunoinhibitor	0.351	2.829E-15
	HSD17B4	HLA-DPB1	MHC	0.345	8.493E-15
	HSD17B4	CD86	Immunostimulator	0.335	5.339E-14
	HSD17B4	TNFSF13B	Immunostimulator	0.322	5.752E-13
LUAD	ACAA1	TNFSF13	Immunostimulator	0.508	1.018E-33
	HSD17B4	TNFSF13	Immunostimulator	0.508	1.092E-33
	HSD17B4	TMEM173	Immunostimulator	0.453	2.341E-26
	PXMP4	TNFSF13	Immunostimulator	0.452	3.008E-26
	HSD17B4	HLA-DMA	MHC	0.426	4.183E-23
	HSD17B4	HLA-DOA	MHC	0.421	1.278E-22
	ACAA1	TNFRSF14	Immunostimulator	0.405	6.396E-21
	HSD17B4	HLA-DPB1	MHC	0.395	6.592E-20
	HSD17B4	HLA-DPA1	MHC	0.391	2.072E-19
	HSD17B4	CD40LG	Immunostimulator	0.390	2.138E-19

Spearman's purity-corrected correlation analysis was used for correlation evaluation and $p < 0.01$ was considered significant. Partial.cor, partial correlation.

Supplementary Table S6. Spearman correlations of HSD17B4, ACAA1 and PXMP4 expressions with IC50 values of the anti-cancer drugs in NSCLC cell lines.

	HSD17B4		ACAA1		PXMP4		
drug	Cor	p	Cor	p	Cor	p	n
17-AAG	-0.135	0.206	-0.074	0.489	0.194	0.068	89
AEW541	0.040	0.709	0.031	0.770	-0.118	0.271	89
AZD0530	-0.161	0.131	-0.261	0.014*	-0.011	0.916	89
AZD6244	-0.065	0.550	-0.281	0.008**	-0.030	0.783	88
Erlotinib	-0.019	0.857	-0.211	0.047*	0.058	0.591	89
Irinotecan	-0.029	0.851	0.098	0.523	-0.133	0.384	45
L-685458	0.155	0.153	0.004	0.970	-0.090	0.409	86
Lapatinib	0.040	0.708	-0.234	0.028*	0.080	0.455	89
LBW242	-0.085	0.429	0.126	0.240	0.090	0.406	88
Nilotinib	0.229	0.053	-0.016	0.894	0.049	0.682	72
Nutlin-3	-0.158	0.140	-0.083	0.439	0.116	0.278	89
Paclitaxel	0.062	0.561	-0.078	0.466	0.018	0.870	89
Panobinostat	0.047	0.665	0.122	0.261	0.076	0.482	87
PD-0325901	-0.060	0.577	-0.196	0.066	0.111	0.299	89
PD-0332991	-0.011	0.927	-0.055	0.646	0.073	0.544	72
PF2341066	-0.149	0.163	0.057	0.596	-0.036	0.737	89
PHA-665752	-0.004	0.968	0.069	0.521	0.023	0.831	88
PLX4720	0.120	0.261	-0.072	0.500	0.064	0.552	89
RAF265	0.056	0.627	0.067	0.558	0.170	0.136	78
Sorafenib	-0.050	0.643	-0.049	0.647	0.073	0.497	89
TAE684	0.006	0.954	-0.098	0.361	-0.052	0.626	89
TKI258	-0.037	0.733	0.109	0.310	0.034	0.753	89
Topotecan	-0.081	0.449	0.149	0.163	0.099	0.354	89
ZD-6474	-0.049	0.652	-0.285	0.007**	-0.079	0.459	89

*,p<0.05; **,p<0.01. Spearman correlation analysis was used and p<0.05 was considered significant.
