# Supplementary material for: HSD17B4, ACAA1, and PXMP4 in Peroxisome Pathway Are Down-Regulated and Have Clinical Significance in Non-small Cell Lung Cancer
Source: Front Genet. 2020 Mar 20;11:273. doi: 10.3389/fgene.2020.00273 (PMC7103649; doi:10.3389/fgene.2020.00273)
Supplement: Supplementary file 2 [file Data_Sheet_2.PDF]

Supplementary Material

2. Supplementary Figures

Supplementary Figure S1 C-DEPGs in LUSC

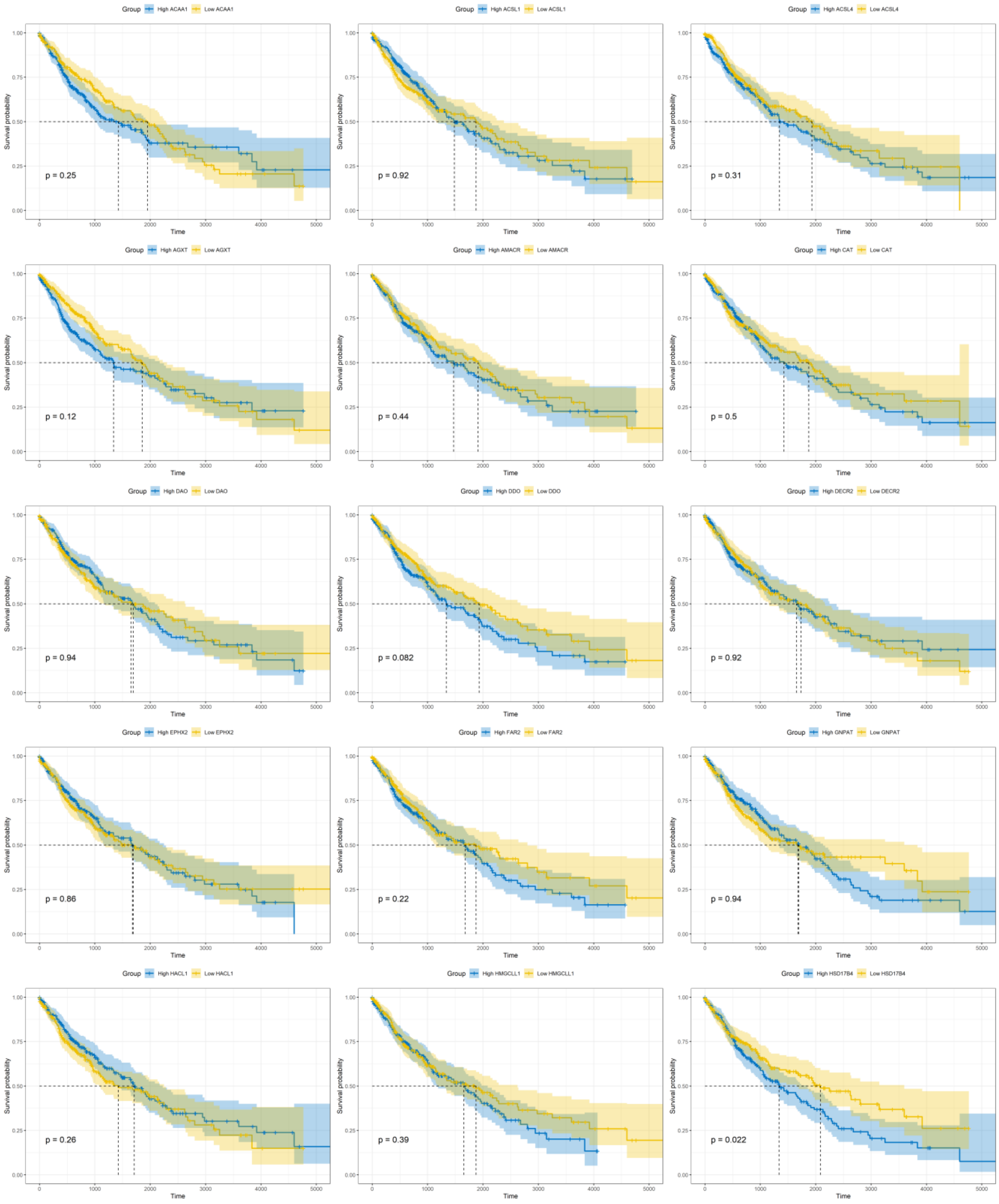

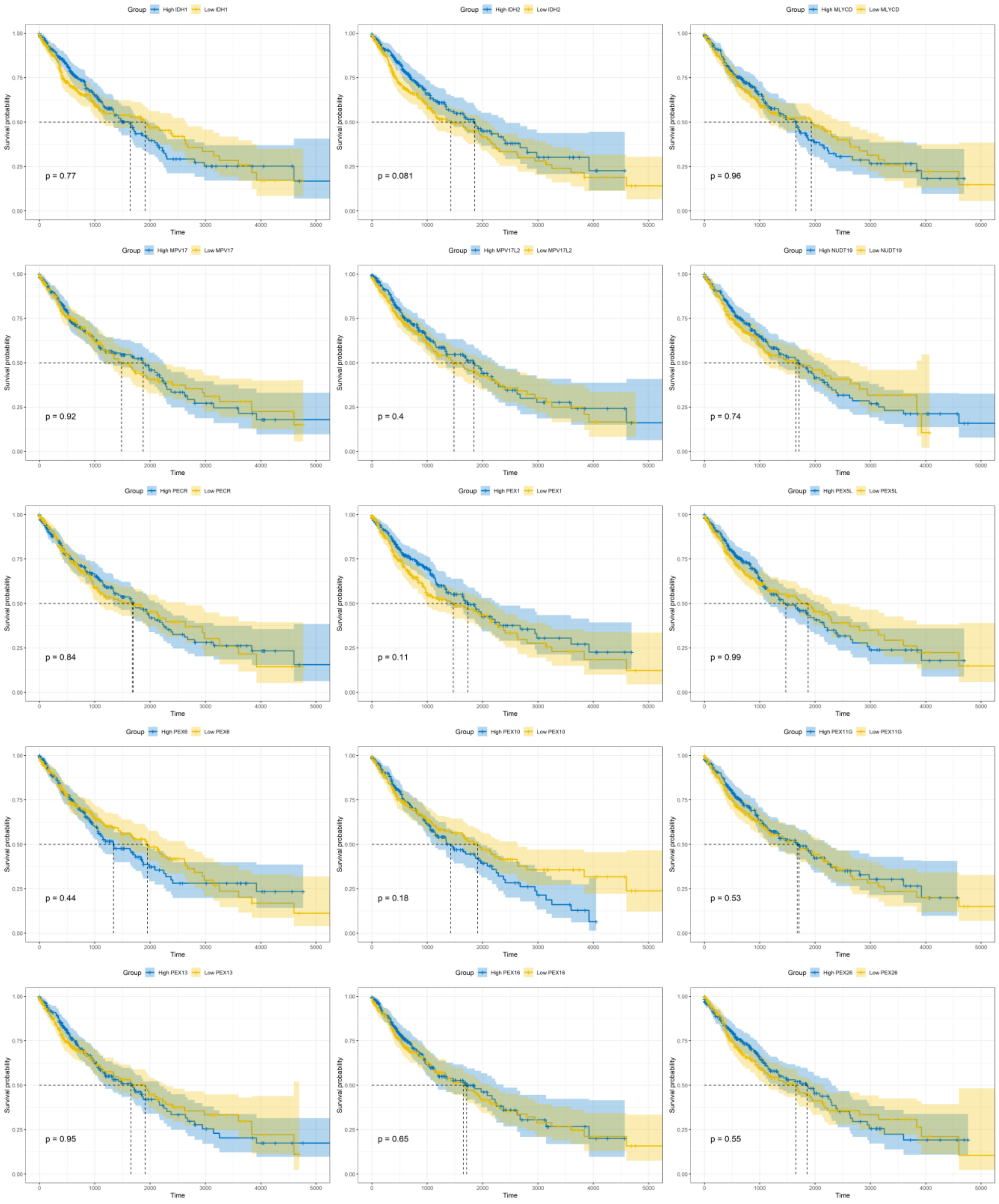
Group High IDH1 Low IDH1
p = 0.77
Group High IDH2 Low IDH2
p = 0.081
Group High MLYCD Low MLYCD
p = 0.96
Group High MPV17 Low MPV17
p = 0.92
Group High MPV17L2 Low MPV17L2
p = 0.4
Group High NUDT19 Low NUDT19
p = 0.74
Group High PECR Low PECR
p = 0.84
Group High PEX1 Low PEX1
p = 0.11
Group High PEX5L Low PEX5L
p = 0.99
Group High PEX6 Low PEX6
p = 0.44
Group High PEX10 Low PEX10
p = 0.18
Group High PEX11G Low PEX11G
p = 0.53
Group High PEX13 Low PEX13
p = 0.95
Group High PEX16 Low PEX16
p = 0.65
Group High PEX26 Low PEX26
p = 0.55
Survival probability
Time

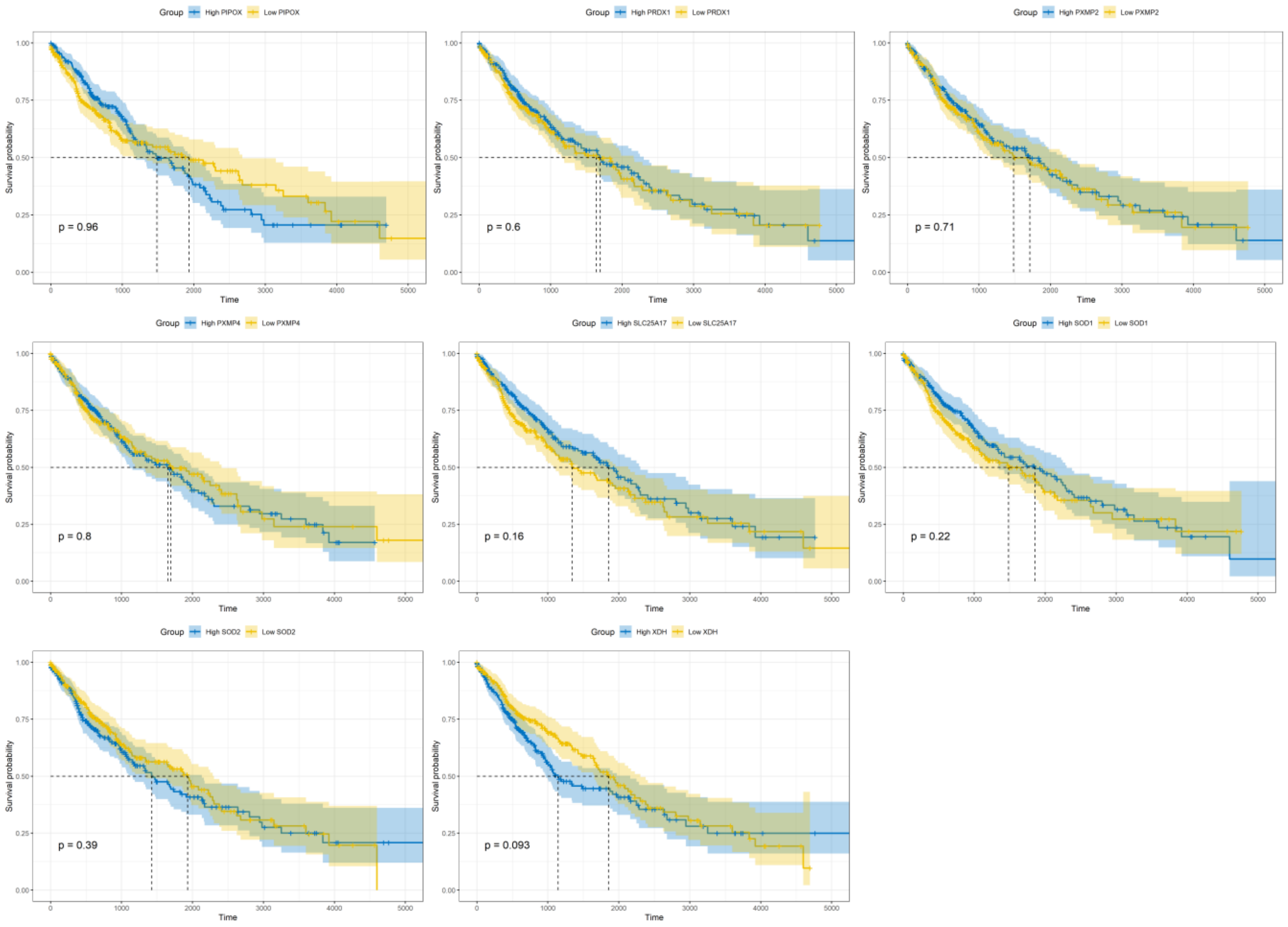

Figure S1. Kaplan-Meier survival analysis of the C-DEPGs in LUSC. The median expression of each gene was set as the threshold for grouping the patients. Kaplan-Meier analysis was used and p<0.05 was considered as statistically significant.

Supplementary Figure S2 C-DEPGs in LUAD

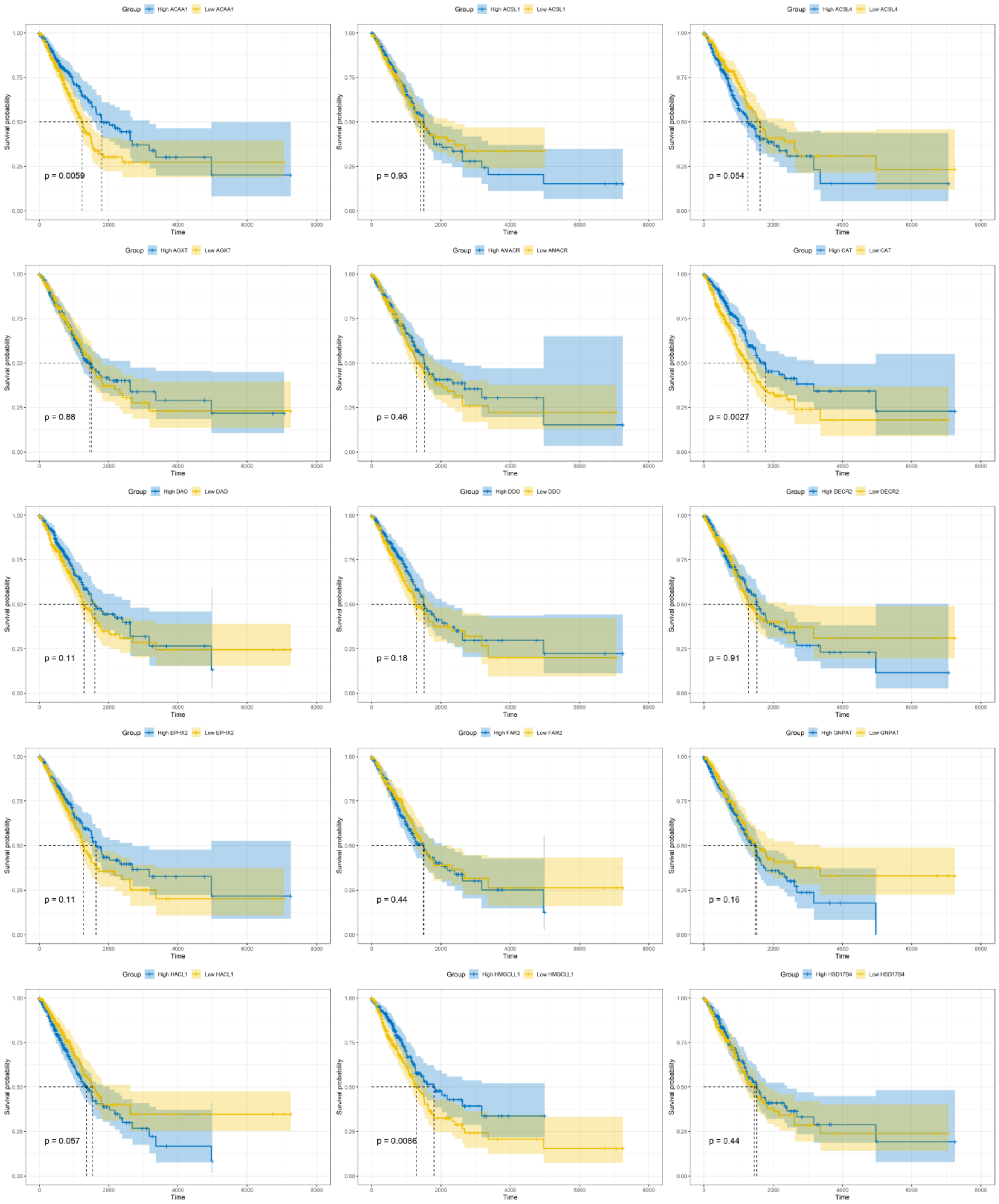

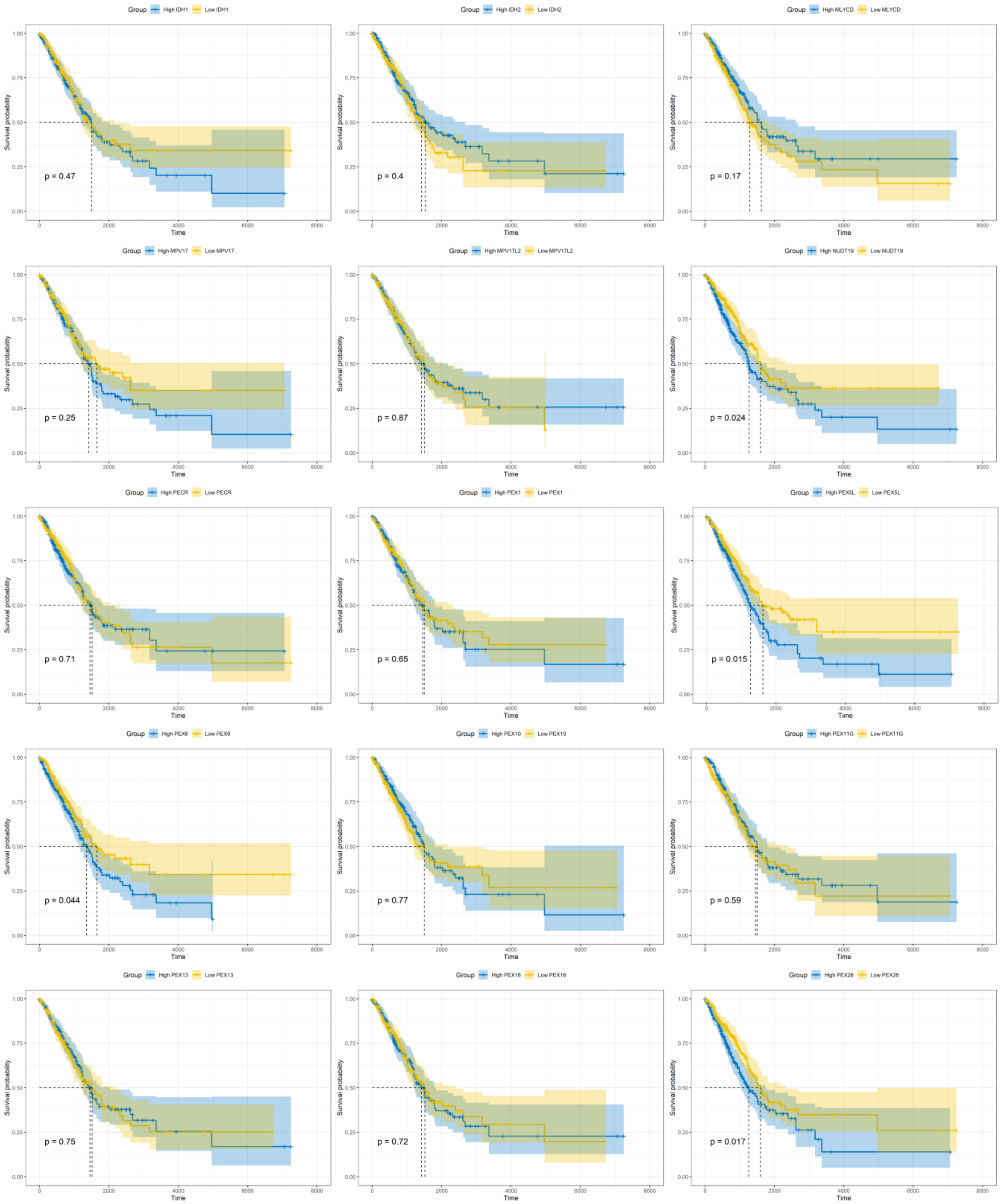
Group High IDH1 Low IDH1
p = 0.47
Group High IDH2 Low IDH2
p = 0.4
Group High MLYCD Low MLYCD
p = 0.17
Group High MPV17 Low MPV17
p = 0.25
Group High MPV17L2 Low MPV17L2
p = 0.87
Group High NUDT19 Low NUDT19
p = 0.024
Group High PECR Low PECR
p = 0.71
Group High PEX1 Low PEX1
p = 0.65
Group High PEX5L Low PEX5L
p = 0.015
Group High PEX6 Low PEX6
p = 0.044
Group High PEX10 Low PEX10
p = 0.77
Group High PEX11G Low PEX11G
p = 0.59
Group High PEX13 Low PEX13
p = 0.75
Group High PEX16 Low PEX16
p = 0.72
Group High PEX26 Low PEX26
p = 0.017
Survival probability
Time

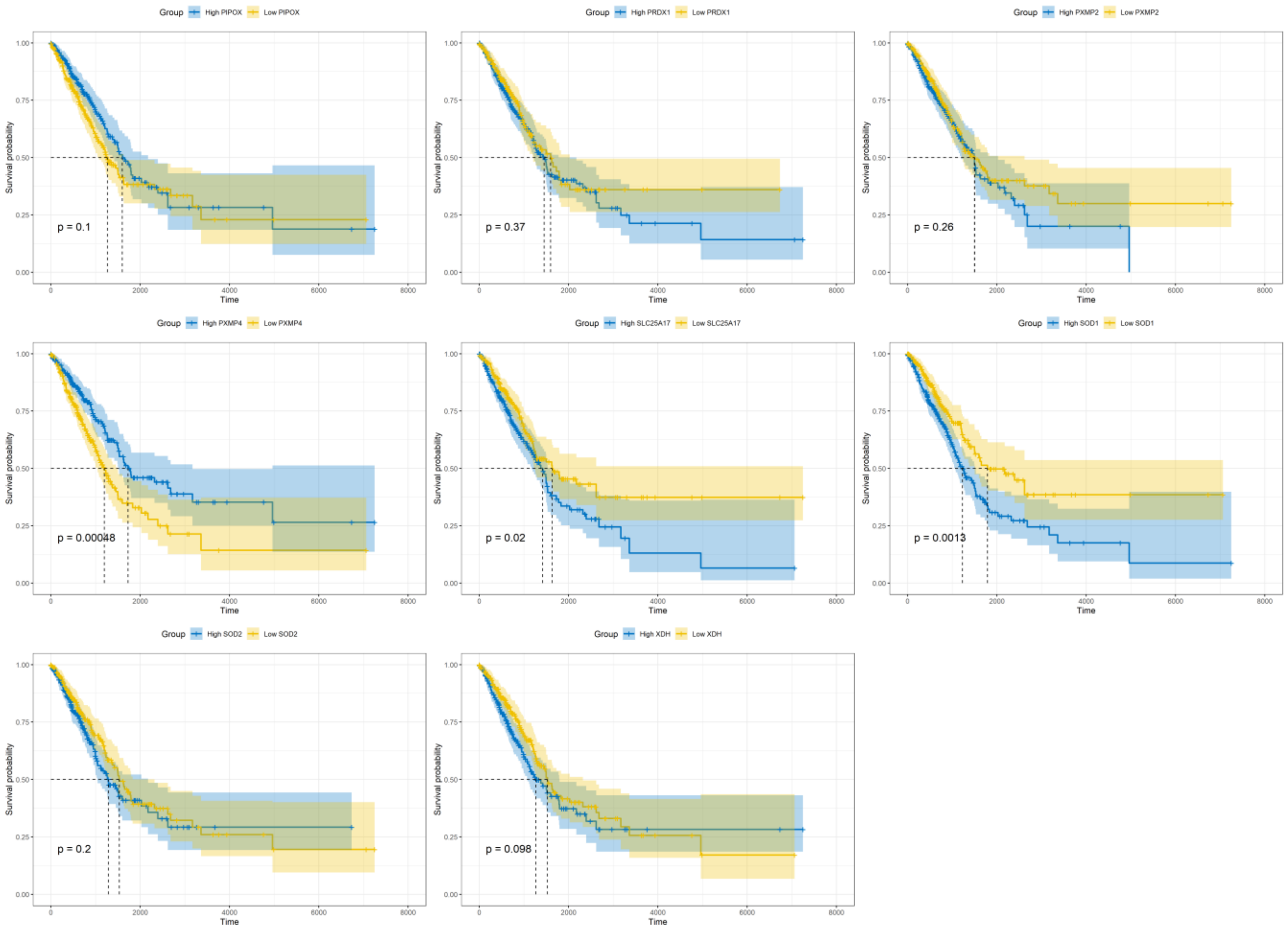

Figure S2. Kaplan-Meier survival analysis of the C-DEPGs in LUAD. The median expression of each gene was set as the threshold for grouping the patients. For the analyses, $p<0.05$ was considered as statistically significant.

Supplementary Figure S3

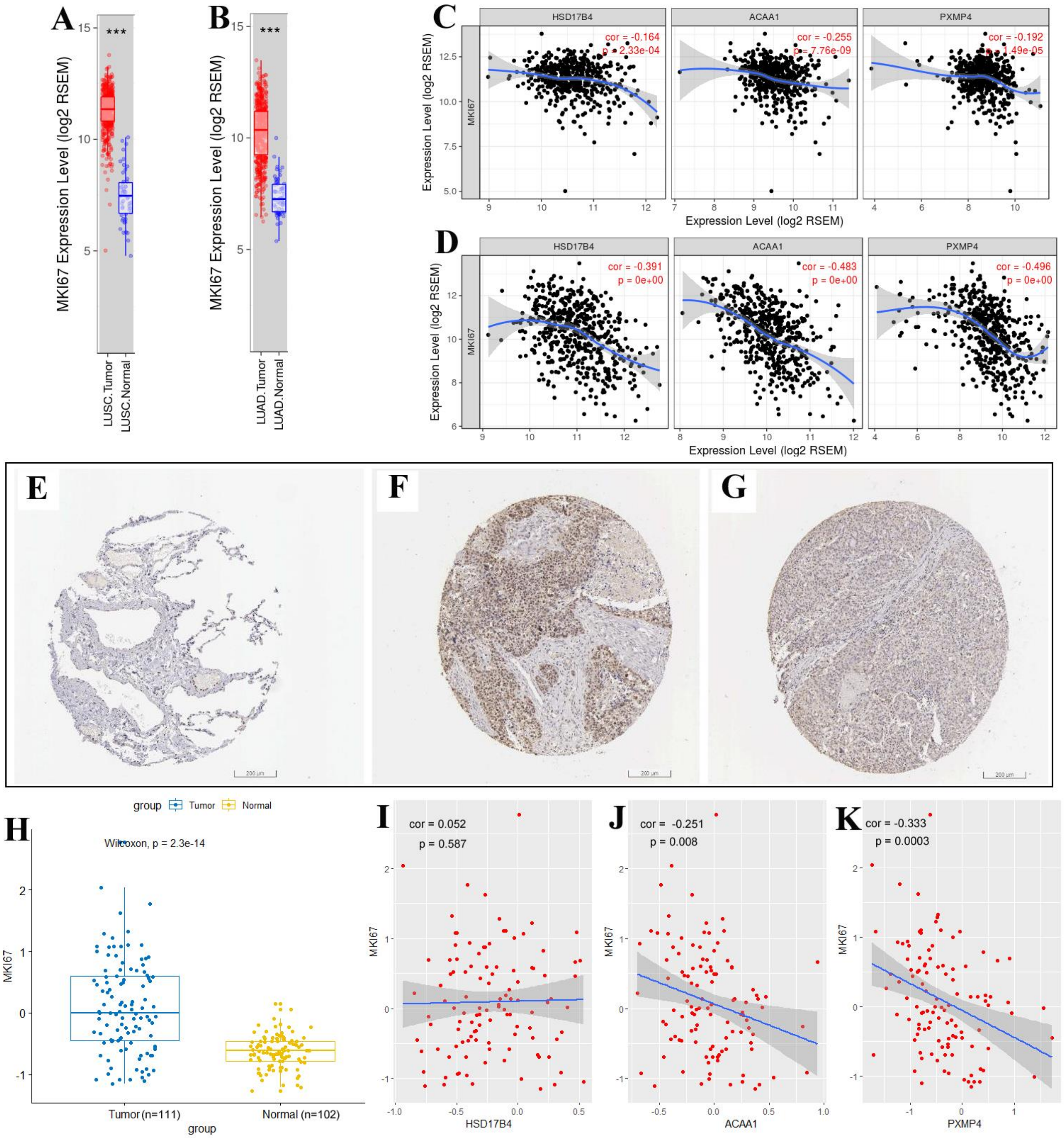

Figure S3. MKI67 expression profiles and its correlations with HSD17B4, ACAA1, and PXMP4 expressions in NSCLC. (A-B) Higher expression of MKI67 in LUSC and LUAD tumors than their normal controls at RNA level. (C-D) HSD17B4, ACAA1, and PXMP4 expressions were negative correlated with MKI67 expression in LUSC and LUAD, respectively. (E) Negative staining of MKI67 in normal lung (not detected in pneumocytes). (F-G) Positive staining (moderate intensity) of MKI67 in LUSC and LUAD tumor cells, respectively. (H) Higher expression of MKI67 in the tumors than the normal controls in the CPTAC-lung cancer dataset. (I-K) Significant negative correlations between

MKI67 expression and HSD17B4, ACAA1, and PXMP4 in CPTAC lung cancer. All the immunostaining pictures (E-G) were downloaded from HPA database and antibody HPA000451was used for staining. NSCLC, non-small cell lung cancer; LUSC, lung squamous carcinoma; LUAD, lung adenocarcinoma; HPA, Human Protein Atlas. CPTAC, Clinical Proteomic Tumor Analysis Consortium. Wilcoxon test and Spearman correlation analysis was used for expressional difference analysis and correlation analysis. $P<0.05$ was considered statistically significant.
